# Structure and Fate of Nanoparticles Designed for the Nasal Delivery of Poorly Soluble Drugs

**DOI:** 10.1021/acs.molpharmaceut.1c00366

**Published:** 2021-07-14

**Authors:** Adryana Rocha Clementino, Giulia Pellegrini, Sabrina Banella, Gaia Colombo, Laura Cantù, Fabio Sonvico, Elena Del Favero

**Affiliations:** † National Council for Scientific and Technological Development—CNPq, Brazilian Government, Brasília DF, 70311-000, Brazil; ‡ Food and Drug Department, University of Parma, Parco Area delle Scienze 27/A, 20090 Parma, Italy; § Department of Medical Biotechnologies and Translational Medicine, LITA, University of Milan, Via Fratelli Cervi 93, Segrate, 20122 Milan, Italy; ∥ Department of Life Sciences and Biotechnology, University of Ferrara, Via Fossato di Mortara 17/19, 44121 Ferrara, Italy; ⊥ Biopharmanet-TEC, University of Parma, Parco Area delle Scienze 27/A, 20090 Parma, Italy

**Keywords:** chitosan, poly-ε-caprolactone, sodium caproyl hyaluronate, nose-to-brain delivery, SANS, nanomedicines

## Abstract

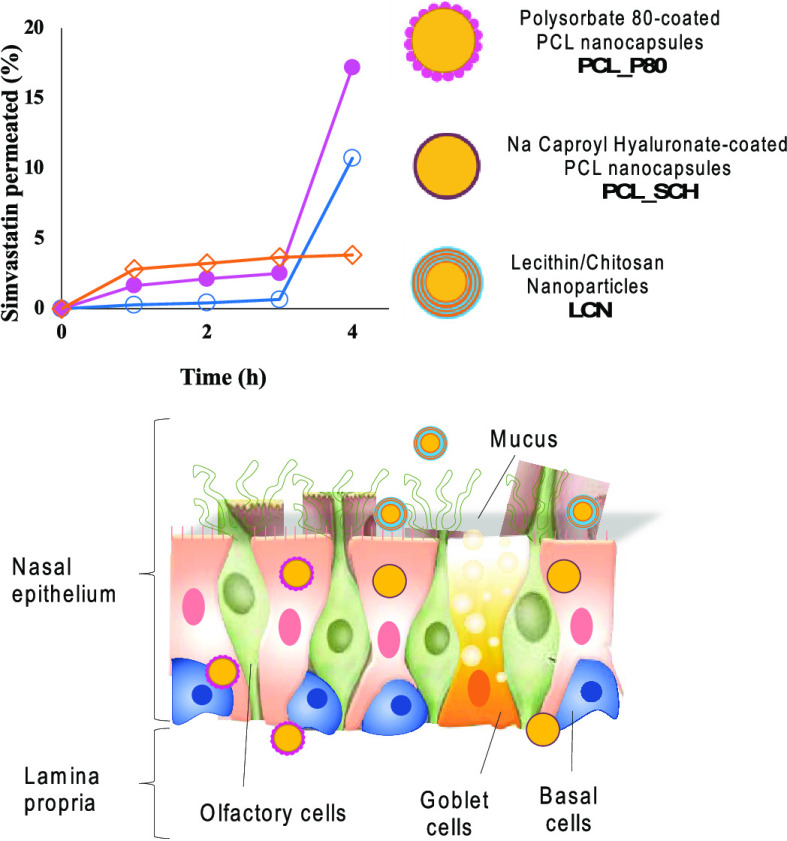

Nanoparticles are promising mediators to enable nasal systemic and brain delivery of active compounds. However, the possibility of reaching therapeutically relevant levels of exogenous molecules in the body is strongly reliant on the ability of the nanoparticles to overcome biological barriers. In this work, three paradigmatic nanoformulations vehiculating the poorly soluble model drug simvastatin were addressed: (i) hybrid lecithin/chitosan nanoparticles (LCNs), (ii) polymeric poly-ε-caprolactone nanocapsules stabilized with the nonionic surfactant polysorbate 80 (PCL_P80), and (iii) polymeric poly-ε-caprolactone nanocapsules stabilized with a polysaccharide-based surfactant, i.e., sodium caproyl hyaluronate (PCL_SCH). The three nanosystems were investigated for their physicochemical and structural properties and for their impact on the biopharmaceutical aspects critical for nasal and nose-to-brain delivery: biocompatibility, drug release, mucoadhesion, and permeation across the nasal mucosa. All three nanoformulations were highly reproducible, with small particle size (∼200 nm), narrow size distribution (polydispersity index (PI) < 0.2), and high drug encapsulation efficiency (>97%). Nanoparticle composition, surface charge, and internal structure (multilayered, core–shell or raspberry-like, as assessed by small-angle neutron scattering, SANS) were demonstrated to have an impact on both the drug-release profile and, strikingly, its behavior at the biological interface. The interaction with the mucus layer and the kinetics and extent of transport of the drug across the excised animal nasal epithelium were modulated by nanoparticle structure and surface. In fact, all of the produced nanoparticles improved simvastatin transport across the epithelial barrier of the nasal cavity as compared to a traditional formulation. Interestingly, however, the permeation enhancement was achieved via two distinct pathways: (a) enhanced mucoadhesion for hybrid LCN accompanied by fast mucosal permeation of the model drug, or (b) mucopenetration and an improved uptake and potential transport of whole PCL_P80 and PCL_SCH nanocapsules with delayed boost of permeation across the nasal mucosa. The correlation between nanoparticle structure and its biopharmaceutical properties appears to be a pivotal point for the development of novel platforms suitable for systemic and brain delivery of pharmaceutical compounds via intranasal administration.

## 1 Introduction

Nasal delivery is emerging as one of the most interesting routes for the systemic delivery of pharmacologically active molecules and a viable alternative to more conventional oral and parenteral administration. In fact, it allows for easy and noninvasive administration, avoids the first-pass metabolism,^1,2^ and potentially provides a direct access to the central nervous system (CNS) bypassing the blood–brain barrier (BBB).^3−5^ In particular, the nose-to-brain (N2B) delivery offers a unique opportunity for drug transport into CNS, taking advantage of the nasal cavity innervation, i.e., the olfactory nerve, connecting the olfactory bulb with the olfactory region of the nasal cavity, and the trigeminal nerve.^6^ Nevertheless, the physicochemical and biopharmaceutical properties of the formulation, as well as the peculiar anatomy and physiology of the nasal route, may hamper drug absorption through the nasal mucosa.^7−9^ In fact, the amount of drug transported from the nose to the systemic circulation is quite variable, ranging from almost 100% to less than 1% of the putative administered dose.^9−11^ In the case of molecules showing low bioavailability after nasal administration, it has been demonstrated that the delivery of therapeutically relevant amounts of drugs is strongly dependent on the availability of efficient formulations and carriers.^9−11^ In recent years, several research groups have shown that nanoparticles constitute a promising strategy to significantly enhance the transport of therapeutics across the nasal mucosa.^12−14^ In particular, nanocapsules, defined as nanosized drug delivery systems having an oily core enclosed by a polymer shell, have been reported to be among the most interesting nanocarriers for drug delivery, providing high drug loading, low polymer content, controlled release rate, efficient protection from degradation factors, and reduced tissue irritation potential.^15^ Very few studies have, however, attempted to elucidate how the drug is actually transported across the mucosa (and even to the brain) by the encapsulating nanoparticles, a missing, but crucial, piece of information. The general concern about the potential toxicity of nanomaterials as drug delivery systems, for example, is of particular relevance if they can enter the CNS level. In fact, in the case of nose-to-brain delivery, nanoparticles could improve drug brain availability in the brain promoting the transport of the encapsulated drug across the neuroepithelium either by a transcellular pathway followed by axonal transport or paracellularly, promoting the diffusion into the brain perineurally or perivascularly.^11,16^ Another aspect to consider is that sometimes the improvement of the nasal transport of encapsulated drugs is due to the shielding of the drug from enzymatic degradation and to diminished nasal clearance, which may occur either by increased retention time or by enhanced carrier permeation across the nasal epithelium.^17−20^ These open issues involve the structural and molecular aspects of nanoparticles, including size, surface properties, and internal arrangement. Altogether, these features are likely to determine the biopharmaceutical behavior of the formulation and in particular how and how efficiently the drug is transported across the biological barriers, such as the nasal mucosa.

Given the general properties of the composing molecules, little is known on whether and how their arrangement, which shapes the supramolecular structure of the nanoparticles, can affect the precise mechanism of interaction with the nasal epithelium and the pathway for drug delivery, leading to different biopharmaceutical responses. The structural properties of the nanoparticles are crucial throughout the life of the drug delivery system, influencing their behavior from the loading of the drug, determining the type of interaction of the formulation with the nasal epithelium, and all the way up to the transport of the drug to the systemic circulation or even the brain. Therefore, in this work, we evaluated the physicochemical and structural properties of the nanoparticles in connection to in vitro and ex vivo biopharmaceutical parameters of nanoformulations proposed as candidates for the nasal and/or nose-to-brain delivery of lipophilic drugs.

Three nanoformulations based on different formulative approaches are considered highly promising for transmucosal drug delivery: a positively charged chitosan-coated lecithin-based nanocarrier (lecithin–chitosan nanoparticles, LCNs)^21,22^ and two poly-ε-caprolactone (PCL)-based nanocapsules, stabilized either with a pegylated surfactant, such as polysorbate 80 (PCL_P80),^23,24^ or with the negatively charged amphiphilic sodium caproyl hyaluronate (PCL_SCH), here used as a novel stabilizer for PCL nanoparticles.

These paradigmatic formulations suitable for the encapsulation of lipophilic drugs and based on lipids, polymers, and surfactants were produced by different manufacturing techniques. In particular, LCNs were obtained by self-assembling to generate a multilayer structure alternating phospholipid bilayers and positively charged polysaccharide chains of chitosan.^25^ Alternatively, PCL, a biocompatible and biodegradable synthetic polymer, was used for obtaining nanocapsule formulations by interfacial polymeric deposition with the nonionic surfactant polysorbate 80 or the hyaluronate derivative sodium caproyl hyaluronate acting as the stabilizer.

Simvastatin (SVT) was selected as a model lipophilic drug, which could benefit from nanoencapsulation, being statins potentially beneficial in the prevention and treatment of several conditions beyond hypercholesterolemia-related cardiovascular diseases, including CNS cancer and neurodegenerative diseases.^26,27^ The use of statins for these pathologies would greatly benefit from a drug delivery approach such as N2B, able to provide a direct delivery to the CNS of the drug, avoiding systemic metabolism and side effects.^28^

A wide set of complementary characterization techniques, ranging from dynamic light scattering (DLS) and small-angle neutron scattering (SANS) for size and structure assessment to experiments at the biological interface, was applied. The objective was to correlate the features of the selected paradigmatic nanoparticles with their impact on the various biopharmaceutical aspects critical for nasal delivery, i.e., their biocompatibility to the site of administration, their interaction with a nasal mucus model, their adhesion strength within the nasal epithelium, their residence time into the nasal cavity, and transport of the SVT model drug across the nasal mucosal epithelium.

## 2 Experimental Section

### 2.1 Materials

Chitosan (Chitoclear FG, 95% deacetylation degree, 105 mPa·s viscosity, and ∼150,000 g/mol MW) was supplied by Primex (Siglufjordur, Iceland) and used without further purifications. Soybean lecithin (Lipoid S45) was obtained from Lipoid AG (Ludwigshafen, Germany). Pharmaceutical-grade oils Labrafac Lipophile WL 1349 (medium-chain triglycerides, EP) and Maisine 35-1 (glycerol monolinoleate) were a kind gift from Gattefossé (Saint-Priest, France). Poly-ε-caprolactone (PCL, MW 14 kDa) was supplied by Fluka-Sigma-Aldrich (St. Louis, MO, USA). Sodium caproyl hyaluronate (MW 200 kDa) was obtained from Contipro Biotech S.r.o. (Dolní Dobrouč, Czech Republic). The surfactants polysorbate 80 (Tween 80) and sorbitan monostearate 60 (Span 60) were purchased from Sigma-Aldrich (St. Louis, MO, USA). Pharmaceutical-grade caprylic/capric triglyceride oil (Miglyol 812) was supplied by Caesar & Loretz GmbH (Mainz, Germany). Simvastatin (MW 418.6 g/mol) was provided by Polichimica (Bologna, Italy). Bovine serum albumin (BSA), mucin from porcine stomach type III (partially purified powder), deuterium oxide (D_2_O, 99.9 atom % D), and dialysis tubing cellulose acetate (14,000 Da molecular weight cutoff, MWCO) were purchased from Sigma-Aldrich (St. Louis, MO, USA). The human nasal septum carcinoma cell line RPMI 2650 (batch CCL-30) was purchased from American Type Culture Collection (ATCC) (Manassas, VA, USA). Minimal essential medium (MEM), fetal bovine serum (FBS), and nonessential amino acid solution were provided by Life Technologies (ThermoFisher Scientific, Waltham, MA, USA). All Transwell cell culture inserts and other consumables were purchased from Corning Inc. Life Science (Corning, NY, USA). Ultrapure water (Purelab Flex, ELGA-Veolia LabWater, Italy) was used in all experiments, except for the specified cases where D_2_O was used. All other chemical reagents were of analytical grade.

### 2.2 Methods

#### 2.2.1 Nanoparticle Preparation

##### 2.2.1.1 Simvastatin-Loaded Lecithin/Chitosan Nanoparticles (SVT-LCNs)

Lecithin/chitosan nanoparticles loading simvastatin (SVT, 1 mg·mL^–1^ final concentration) were prepared following a previously reported protocol.^22^ Briefly, SVT was dissolved in a lecithin alcoholic solution (2.5% w/v) containing Maisine and Labrafac oils (1:1) that was injected into a chitosan aqueous solution (0.01% w/v). Blank nanoparticles were produced as well, omitting simvastatin from the organic phase (see the Supporting Information for the detailed protocol).

##### 2.2.1.2 Simvastatin-Loaded PCL Nanocapsules (SVT-PCL_P80 and SVT-PCL_SCH)

Blank and SVT-loaded poly-ε-caprolactone nanocapsules were prepared adapting an interfacial polymeric deposition methodology reported previously.^29,30^ In brief, PCL, SVT, caprylic/capric triglyceride oil, and sorbitan monostearate 60 were dissolved in 5 mL of acetone. Then, 1 mL of a 0.06% w/v lecithin ethanol solution was added to complete the organic phase. The aqueous phase was obtained by dissolving polysorbate 80 into 10 mL of ultrapure water (0.076% w/v). SVT-PCL_P80 nanoparticles were then formed by polymeric nanoprecipitation following injection of the organic phase into the aqueous solution, under magnetic stirring at 40 °C. The organic solvents were evaporated using a rotary evaporator (Heidolph WB/VV 2000, Schwabach, Germany) set at 40 °C, and the formulation was further concentrated to a final volume of 10 mL (see the Supporting Information for the detailed protocol).

The production of blank and SVT-loaded sodium caproyl hyaluronate (SVT-PCL_SCH) nanoparticles was carried out viaof the same methodology using an aqueous phase containing sodium caproyl hyaluronate acting as surfactant and stabilizer. All PCL-based nanocapsules were produced in triplicate, at least.

#### 2.2.2 Nanoparticle Physicochemical Characterization

##### 2.2.2.1 Nanoparticle Size and ζ-Potential Determination

Nanoparticle diameter, polydispersity index (PI), and ζ-potential (ZP) were determined using a Malvern Zetasizer Nano ZSP (Malvern Instruments Ltd., Malvern, U.K.).

Nanoparticle diameter and PI were measured using dynamic light scattering (DLS) at a 173° scattering angle. Prior to measurements, each formulation, i.e., blank and simvastatin-loaded nanoparticles, was diluted (1:100) with distilled water filtered with 0.22 μm filters (mixed cellulose ester membrane, Merck Millipore, Burlington, MA) to avoid multiple scattering. For DLS measurements, the instrument was operated at 25 °C . Three measurements were recorded for all nanoparticles. ZP was determined through phase analysis light scattering (PALS) using the same diluted samples prepared for particle size analysis. ZP values are presented as mean and standard deviation of three separated runs for each sample prepared in triplicate.

##### 2.2.2.2 Drug Encapsulation Efficiency of Nanoparticles

SVT content and encapsulation efficiency (EE%) were evaluated by a direct and an indirect method by high-performance liquid chromatography (HPLC), respectively, following a previously published protocol.^22,31^ Encapsulation efficiency was expressed as percentage of encapsulated drug with respect to the total amount present in the formulation. Briefly, the total amount of SVT (total SVT in eq 1) in each formulation batch was determined by directly dissolving 100 μL of SVT-loaded samples into 10 mL of standard diluent (ethanol/acetonitrile/water, 55:30:15, v/v/v, pH 4.5). On the other hand, the free, i.e., nonencapsulated, drug (free SVT in eq 1) in the preparation was determined by ultrafiltration using the Vivaspin centrifugal concentrator (PES, MWCO 30 kDa, Sartorius, Gottingen, Germany). The encapsulation efficiency of SVT in nanoparticles was then calculated using the following equation:
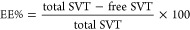
1All quantification analyses were performed following the HPLC protocol reported in Section 2.2.2.3. Refer to the Supporting Information for the detailed protocol.

##### 2.2.2.3 High-Performance Liquid Chromatography Method for the Determination of SVT in Nanoparticles

The SVT content in nanoparticles was measured using an already published and validated high-performance liquid chromatography (HPLC) protocol.^31^ The full method description and validation are presented in the Supporting Information.

#### 2.2.3 Nanoparticle Structure and Interaction with Simulated Nasal Mucus

The internal structure of nanoparticles and their structural modifications upon interaction with a nasal mucus model can be investigated by advanced scattering techniques.^32,33^ X-rays or neutron scattering experiments allow for a description of the structure of the nanoparticles down to the length-scale of the incident radiation wavelength. We performed small-angle neutron scattering (SANS) experiments (accessible length range: from 2 to 300 nm) at the D33 beamline of the Institute Laue-Langevin (ILL, Grenoble, France)^34^ at *T* = 25 °C. All nanoparticle batches and simulated nasal mucus used in SANS experiments were produced using D_2_O instead of water in the preparation process to allow for a liquid environment with proper contrast for neutron scattering.^35^ Spectra were obtained at two different sample-to-detector distances (2 and 12 m) and then carefully joined after normalization and background subtraction. SANS profiles report the scattered intensity as a function of the momentum transfer *q* (see eq 2):
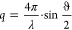
2where θ is the scattering angle and λ = 8 nm is the incident neutron wavelength. The investigated *q*-range was 2 × 10^–3^ Å < *q* < 0.3 × 10^–1^ Å. For a population of noninteracting nanoparticles, the intensity *I*(*q*) is proportional to the form factor *P*(*q*) of particles. SANS profiles were reconstructed with the SasView application (version 4.2.0, 2019).

To assess the structural stability of the nanoparticles in the presence of simulated nasal mucus, SANS experiments were repeated on the same formulations after addition of a simulated nasal mucus, containing 0.5% w/v porcine mucin in a simulated nasal electrolyte solution (SNES: 8.77 mg·mL^–1^ sodium chloride, 2.98 mg·mL^–1^ potassium chloride, and 0.59 mg·mL^–1^ calcium chloride aqueous solution),^36^ upon 15 min of incubation. For each incubated system, SANS profiles were analyzed by subtracting the mucus spectrum and comparing the remaining signal with the profiles obtained with the corresponding nanoparticles before mucus incubation.

#### 2.2.4 In Vitro Cytotoxicity Assay of Nanoparticles

Cytotoxicity of empty nanoparticles against the human nasal cell line RPMI 2650 was performed using the 3-(4,5-dimethylthiazol-2-yl)-2,5-diphenyltetrazolium bromide (MTT) colorimetric assay after 72 h of nanoparticle incubation with the cells. RPMI 2650 cells were routinely cultured in MEM containing 10% v/v FBS and 1% nonessential amino acid solution and incubated at 37 °C with 95% air humidity and a 5% CO_2_ atmosphere.^37^ For the cytotoxicity experiment, 5 × 10^4^ cells per well were seeded in a 96-well cell culture plate (Corning Life Science, Tewksbury, MA) and incubated for 24 h to allow for cell adhesion. Then, to evaluate nanoparticle cytotoxicity, the same amount of blank nanoparticles was tested for all three formulations (0–610 μg·mL^–1^ w/v) by diluting the freshly prepared nanoparticle suspension (LCN 6.1 mg, PCL_P80 9.06 mg, and PCL_SCH 6.86 mg initial concentration) at least 1:10 with the culture medium. After 72 h of incubation, the cells were washed with phosphate-buffered saline (PBS) and incubated for 2 h with MTT reagent (5 mg·mL^–1^). Then, the MTT medium was removed, and 120 μL of dimethyl sulfoxide (DMSO) was added to each well to dissolve the violet-colored formazan metabolite formed by enzymatic oxidoreduction of the tetrazolium dye in the mitochondria of viable cells. Spectrophotometric absorbance for each well was measured at 570 nm using a microplate reader (Spark 10M, Tecan, Männedorf, Switzerland), and values were considered directly proportional to cell viability. Formulation toxicity was represented as the percentage of nasal cell survival after treatment taking as reference the values obtained for untreated cells. Experiments were performed in triplicate on different days and cell passages (between 18 and 22 passages).

#### 2.2.5 Simvastatin Release from Nanoparticles

*In vitro* release studies of SVT from drug-loaded LCN, PCL_P80, and PCL_SCH nanoparticles were carried out applying the dialysis bag diffusion method.^38^ SNES was used as the dissolution medium since these nanoparticles are intended for nasal administration. Bovine serum albumin (BSA, 0.5% w/v) was used to increase SVT solubility in SNES (from 25 to 52 μg·mL^–1^).^22^ For each formulation, 1 mL of the nanoparticle suspension (corresponding to ∼1 mg of SVT) was dispersed into 1 mL of SNES + BSA 0.5% w/v, pH 6.5, to mimic nasal physiological conditions. Each 2 mL dispersion was separately placed in the dialysis tubing cellulose membrane (MWCO 14 kDa, Sigma-Aldrich, St. Louis, MO, USA), in triplicate. The sealed bags were immersed into a graduated glass cylinder containing 100 mL of the dissolution medium (SNES + BSA 0.5% ), kept at 37°C and magnetically stirred at 100 rpm. At predetermined time points (1, 2, 3, 4, 5, 6, 7, 8 and 24 h), 500 μL aliquots of the dissolution medium were withdrawn from the cylinder. The sampled volume from each cylinder was replaced with an equal volume of fresh dissolution medium. Samples were then pretreated with 25 μL of concentrated perchloric acid to precipitate and remove BSA by centrifugation (10 min at 21 380*g*; D3024 Microcentrifuge, Scilogex, Rocky Hill, CT, USA). The obtained supernatants were diluted fourfold in a standard diluent (ethanol/acetonitrile/water adjusted to pH 4.5 with 1.0 M orthophosphoric acid, 55:30:15, v/v/v) and assayed by HPLC to quantify the released SVT. Finally, to calculate the SVT mass balance, the total content of each dialysis bag was quantitatively collected, dispersed into 50 mL of standard diluent, and sonicated for 30 min to extract and quantify the residual drug from the nanoparticulate formulations. All *in vitro* release studies were conducted in triplicate for each formulation and the results reported as percentage of drug released relative to that from the total amount of simvastatin quantified in each experiment.

#### 2.2.6 Nanoparticle Mucoadhesion on Excised Porcine Nasal Epithelium

The bioadhesion properties of chitosan-coated LCN nanoparticles, PCL_P80, and SCH-coated PCL nanocapsules were determined by a previously reported method first introduced by Rao and Buri,^39^ namely, continuous flow assay, to evaluate the extent of adhesion/retention of drug delivery systems on the surface of a mucosal tissue subjected to a controlled gravitational force and continuous wash.

Freshly excised piglet nasal mucosa discs (6 mm diameter, Department of Veterinary Medicine, University of Parma, Italy) were placed by means of double-sided adhesive tape on a glass plate. Then, 10 μL of SVT-loaded LCN, PCL_P80, PCL_SCH nanoparticles, or simvastatin aqueous suspension was applied on the nasal mucosa. After 5 min, the glass plate was positioned on a polystyrene support oriented at a 45° angle from the benchtop and washing of the nasal mucosal surface with SNES was started at constant flow rate (100 μL·min^–1^) for 20 min (syringe pump Model 200, KD Scientific, Holliston, MA, USA). Samples of the eluted washing SNES were collected every 2 min and diluted in the standard diluent and assayed for SVT content by HPLC. At the end of the experiment, each nasal mucosa disc was collected and homogenized with 1 mL of standard diluent to extract and quantify the residual drug still present on or within the mucosal membrane. Results of the mucoadhesion analysis are reported as the amount of residual simvastatin adhering to the nasal mucosa at different washing times, expressed as a percentage of the total SVT recovered for each sample. In addition, a mucosal mean residence time (mMRT) was calculated from the data collected, applying eq 3, adapted from a classic method to calculate the mean residence time in pharmacokinetics:^40^
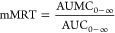
3where AUC is the area under the curve describing the percentage of residual SVT adhering to the tissue vs time and AUMC is the area under the first moment curve. The AUC and AUMC were calculated by the trapezoidal method with exponential extrapolation, and these were used to calculate the MRT. The experimental setup (Figure S1) and an extended description of the protocol are available in the Supporting Information.

#### 2.2.7 Simvastatin Transport across Excised Rabbit Nasal Mucosa

To investigate nanoparticle permeation enhancement properties, rabbit nasal mucosa was obtained from a local slaughterhouse (Pola S.r.l., Finale Emilia, Italy). The experimental conditions were strictly controlled to limit tissue damage and alteration.^41^ The nasal mucosa specimens were rinsed with SNES (pH 6.5) and immediately mounted on vertical Franz-type diffusion cells (Vetrotecnica S.r.l., Padova, Italy; 0.58 cm^2^ permeation area) with the mucosal side facing the donor compartment and the serosal side facing the receptor. For each cell, the receptor chamber was filled with 5 mL of SNES (pH 6.5) and the assembled Franz cell was equilibrated at 37 °C for half an hour in a thermostatic water bath. Thereafter, the SNES solution was removed from the donor compartment and replaced with 1 mL of 1 mg·mL^–1^ freshly prepared nanoparticle formulations, either SVT-loaded LCN, SVT-PCL_P80, or SVT-PCL_SCH. The SVT suspension (1 mg·mL^–1^) in SNES was used as the control. Experiments lasted for 4 h, under constant magnetic stirring of the receptor compartment (800 rpm), to avoid boundary saturation on the mucosal membrane. At predetermined time points (0, 60, 120, 180, and 240 min), aliquots of 500 μL were sampled from the receptor compartment and replaced with the same volume of preheated SNES medium. Samples were kept at −20 °C until analysis.

At the end of the experiment, to calculate the mass balance, donor samples were quantitatively collected, and the compartment was rinsed thoroughly with SNES to recover any formulation residue adhering to the glass walls or the mucosal surface of the nasal tissue. Samples collected from the donor were assayed for SVT content by dissolving 100 μL into 10 mL of acetonitrile/25 mM PBS buffer (65:35 v/v, pH 4.5) and sonicating for 45 min (ultrasonic cleaner; VWR, Radnor, PA, USA) to extract all the drug from the nanoparticles. Extraction and quantification of SVT inside the mucosa were as described in Section 2.2.6. SVT permeation was expressed as the amount permeated per unit area (μg·cm^–2^). A protocol reporting more details on this experiment is available in the Supporting Information.

#### 2.2.8 Statistics

All results are reported as mean value and standard deviation of at least three replicates, if not stated otherwise. All statistics analyses were performed using Prism Software Version 8.0a (Prism, Version 8.0a, GraphPad Software Inc., La Jolla, CA, USA). Data dispersion was verified using one-way analysis of variance (ANOVA) with the post hoc Šídák test for multiple comparisons, considering significant differences with **p < 0.01 and ***p < 0.001 (Prism, Version 8.0a, GraphPad Software Inc., La Jolla, CA).

## 3 Results

### 3.1 Nanoparticle Preparation and Physicochemical Characterization

Although the exact mechanism of nasal delivery of nanoencapsulated drugs is still debated, the improved availability of intranasally administered drugs induced by pharmaceutical nanotechnologies is an accepted concept. Delivery efficiency seems strongly dependent on the nanoparticle properties because of increased residence time in the nasal cavity, increased efficiency of drug release, and improved bioavailability through the enhancement of transport of active ingredients across biological membranes.^42^ High biocompatibility is also required to avoid local and systemic toxic effects. However, the interrelation between nanoparticle characteristics and drug transport through the nasal mucosa still remains to be elucidated. Moreover, in the literature, it is very difficult to find studies reporting direct comparisons between drug delivery systems with different physicochemical features and consequently different biopharmaceutical behaviors.

In this work, we evaluated the physicochemical properties and nanoparticle structure of three paradigmatic nanoformulations for the administration of lipophilic drugs, intended for nasal delivery, in connection to their performances in a set of *in vitro* and *ex vivo* experiments. All nanoparticles produced were loaded with simvastatin as a model drug. Simvastatin is a biopharmaceutical Class II drug, presenting poor aqueous solubility and acceptable permeability through biological membranes.^43^ SVT-loaded hybrid lecithin/chitosan nanoparticles (SVT-LCNs) were obtained by a spontaneous self-assembly process, involving the electrostatic interaction of lecithin, a negative phospholipid, with chitosan, a positively charged polysaccharide.^44,45^ These nanosystems combine the versatility of phospholipid-based nanocarriers with the penetration-enhancing properties of positively charged polysaccharide chitosan and have been demonstrated to be promptly biodegradable.^22,46^ Lipid-core PCL nanocapsules were obtained using either a nonionic surfactant, i.e., polysorbate 80 (PCL_P80),^29^ or a negatively charged polysaccharide-based surfactant, i.e., sodium caproyl hyaluronate. Polysorbate 80 is a pegylated nonionic surfactant often used to stabilize the nanoparticle surface, which, besides providing a longer circulation time to intravenously administered nanocarriers, has been regularly reported to be able to increase brain delivery also via several others administration routes, such as oral^47^ and nasal.^48^ Hyaluronic acid and its derivatives have been proposed as nanoparticle coating materials since this polysaccharide is highly biocompatible, provides a hydrophilic “corona” to the particles, and allows particle endocytosis in CD44 receptor expressing cells, as in the case of several tumors.^49,50^

The physicochemical properties of SVT-loaded LCN, SVT-PCL_P80, and SV-PCL_SCH nanoparticles and their corresponding blank formulations are shown in Table 1.

**Table 1:** Nanoparticle Physicochemical Properties

**Batch**	**Size (nm)**	**PI**	**ZP (mV)**	**EE%**
blank LCN	187.6 ± 6.8	0.10 ± 0.01	+48.5 ± 2.0	-
SVT-LCN	212.6 ± 7.2	0.11± 0.06	+40.4 ± 2.1	99.3 ± 1.1
blank PCL_P80	140.3 ± 10.8	0.10 ± 0.03	–14.2 ± 0.6	-
SVT-PCL_P80	202.5 ± 18.0	0.12 ± 0.08	–22.2 ± 3.2	99.8 ± 0.7
blank PCL_SCH	255.1 ± 9.0	0.10 ± 0.01	–34.6 ± 5.0	-
SVT-PCL_SCH	258.0 ± 7.9	0.15 ± 0.04	–39.2 ± 7.0	97.3 ± 1.3

All formulations were highly reproducible, showing relatively small (∼200 nm) and narrowly distributed (PDI < 0.2) nanoparticles. However, the use of different ingredients and/or preparation methods influenced the nanoparticle structure and surface charge, as seen from the measured ζ-potential. For instance, chitosan-coated nanoparticles presented a highly positive ZP, whereas both PCL-based nanocapsules were negatively charged, with SCH-coated nanoparticles displaying a higher negative ZP compared to the PCL_P80 formulation (−34 mV vs −14 mV), likely due to the contribution of hyaluronate coating on PCL nanocapsules.^51,52^

The introduction of simvastatin in the composition of all three formulations did not substantially modify the physicochemical properties of the corresponding blank nanosystems, even if drug-loaded nanoparticles showed a larger size. Notably, as presented in Table 1, all polymeric nanoparticles, even if produced employing different techniques and/or excipients, promoted the encapsulation of almost the total drug content (up to 97%, 1 mg·mL^–1^), providing a 40-fold increase in SVT solubility in an aqueous environment (25 μg·mL^–1^, as determined experimentally in-house), suggesting an optimized accommodation of the active compound into the hydrophobic core of the nanoparticles.

### 3.2 Nanoparticle Structure and Interaction with a Simulated Nasal Mucus

The internal structure of nanoparticles can be investigated by the small-angle neutron scattering (SANS) technique, performing measurements on blank and SVT-loaded formulations. Moreover, to study their physical stability in the biological medium, the structural alterations, if any, induced in nanoparticles by contact with the nasal mucus model were monitored. Hence, neutron scattering experiments were repeated on the same formulations after 15 min of incubation in a simulated nasal fluid containing mucin (0.5% w/v).

#### 3.2.1 Lecithin/Chitosan Nanoparticles

**Figure 1:**
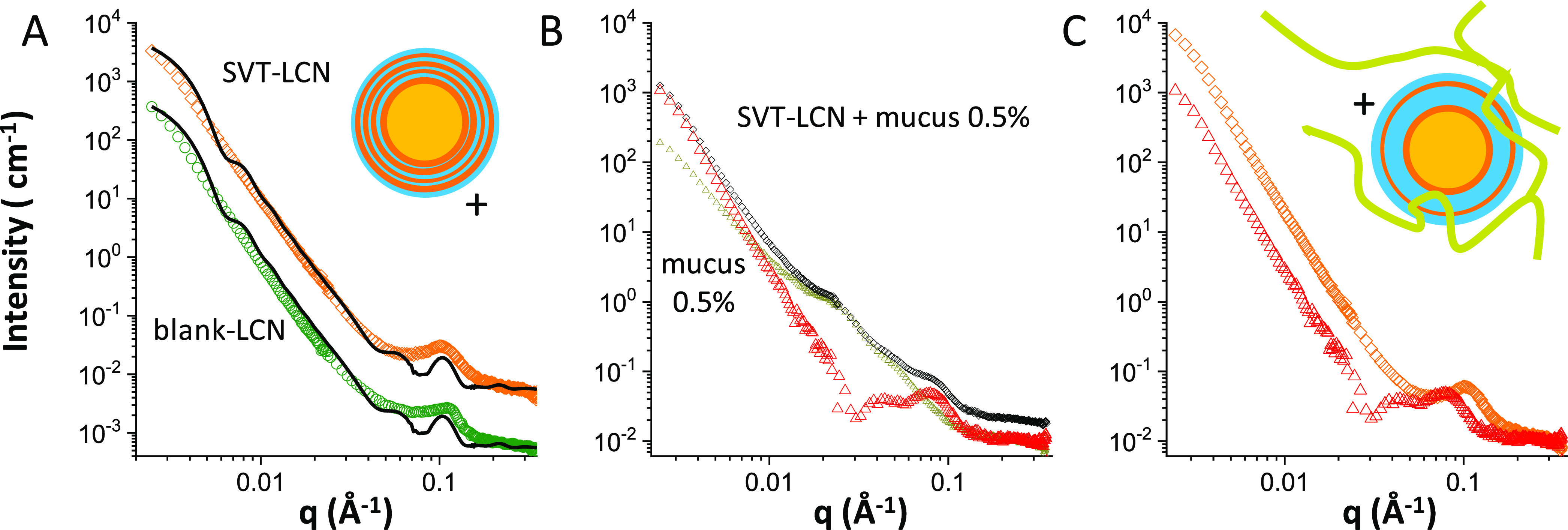
SANS intensity spectra of lecithin/chitosan nanoparticles (8 mg·mL^–1^): (A) spectra of blank LCN (green circles, down-shifted for better visibility) and SVT-LCN (orange diamonds) nanoparticles together with the fit to a spherical core multilamellar–shell model (continuous line); (B) spectra of SVT-LCN dispersed in simulated nasal mucus (porcine mucin 0.5% w/v in SNES) (black diamonds), simulated nasal mucus alone (dark-yellow triangles), and spectrum obtained by subtracting the mucus contribution from the spectrum of nanoparticles in mucus (red triangles); (C) spectra LCN nanoparticles before (orange diamonds) and after (red triangles) interaction with mucus are reported together for better visual comparison.

Figure 1A reports the scattered intensity profiles of both blank (blank LCN) and SVT-loaded (SVT-LCN) LCN nanoparticles. The SANS spectra of both LCN nanoparticles are very similar and show the features typical offor globular structures. The intensity decay in the *q* < 0.02 Å^–1^ region is *I*(*q*) ÷ *q*^–4^, characteristic for particles with a well-defined interface, while in the high *q* region, a structure peak at *q* = 0.1 Å^–1^ reveals a multilamellar layering of the lecithin/chitosan shell. The particle form factor (full lines in the plot) is that expected for a spherical oil-core, 180–200 nm in size, surrounded by a multilamellar shell with interlamellar distance of 6 nm, typical for stacks of lecithin bilayers. The introduction of simvastatin did not alter significantly the internal structure of the particles or the thickness of the shell layer.

To investigate the structural alterations, if any, following nanoparticle interaction with nasal mucus, SANS analyses were carried out on SVT-LCN dispersion added to simulated nasal fluid containing 0.5% w/v porcine mucin. Figure 1B reports the scattered intensity profile of SVT-LCN after 15 min interaction with the simulated mucus. The profile of the simulated mucus alonemodel by itself is also plotted for comparison and displays the characteristic behavior of the mucin chains in simulated nasal fluid.^33^ The mucus-model contribution was then subtracted from the spectrum of the mixed system, and the remaining profile was compared with the one of SVT-LCN nanoparticles before mucus interaction to detect whether structural changes occurred in the nanoparticles (Figure 1C). Spectra show that, upon interaction with mucus, the core/multilamellar–shell structure of the original particles was retained, although the observed decrease in the overall intensity suggests that few lipid bilayers might peel off from the external surface of the multilamellar shell. Moreover, as visible in Figure 1C, the evident down-shift of the multilamellar peak from *q* = 0.1 to 0.089 Å^–1^ reveals that the adjacent bilayers of the external shell swelled to a larger interlamellar distance (7–8 nm). Overall, these data indicate that SVT-loaded LCN nanoparticles were stretched by the presence of the mucus matrix, possibly due to the electrostatic interaction and hydrogen bonding betweenamong chitosan, the coating nanoparticle surface, and the negatively charged glycoproteins (sialic acids and ester sulfates) constituting 90% of the mucin macromolecules.^53−55^ The mucoadhesion of chitosan-coated nanoparticles has been already reported by us and other groups and represents one of the hallmarks of chitosan-based delivery systems.^10,16,56−58^

#### 3.2.2 Polysorbate 80 Stabilized Poly-ε-Caprolactone Nanocapsules

**Figure 2:**
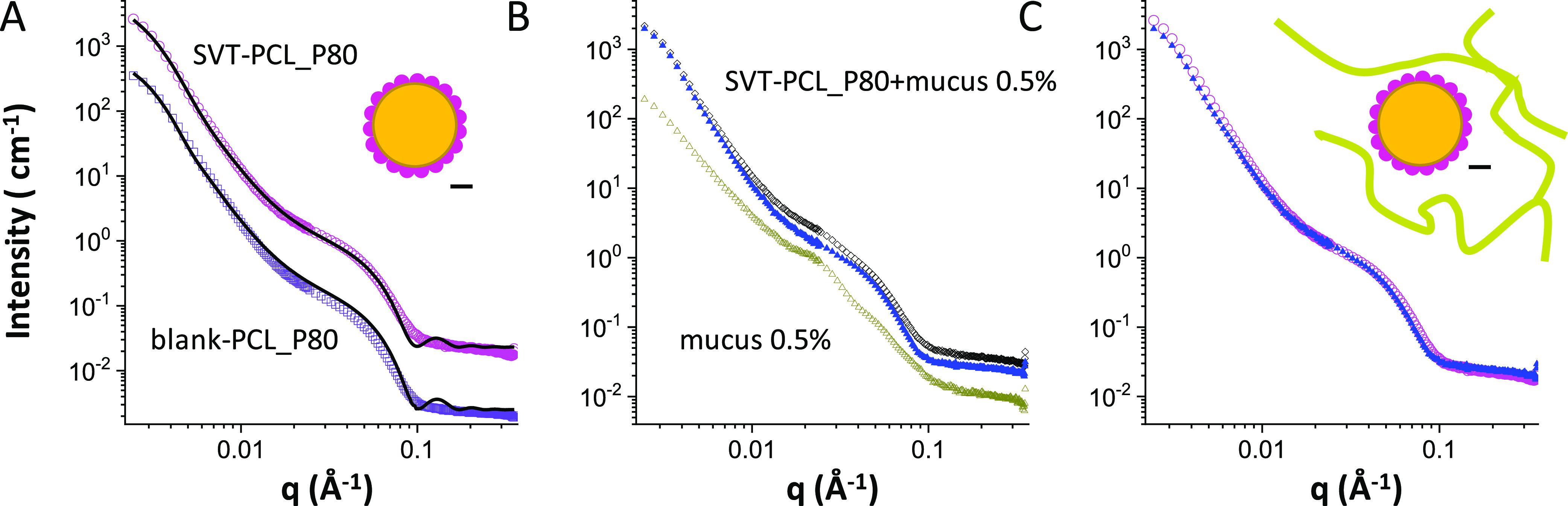
SANS intensity spectra of poly-ε-caprolactone nanocapsules (8 mg·mL^–1^). (A) Loaded SVT-PCL_P80 nanocapsules (magenta dots) and blank-PCL_P80 nanoparticles (violet squares) shifted for better visibility, together with the fits (black lines) to a raspberry model, as sketched. (B) SVT-loaded PCL_P80 dispersed in simulated nasal mucus (porcine mucin 0.5% w/v in SNES) (black diamonds) and simulated nasal mucus alonemucus at 0.5% w/v (dark-yellow triangles). Blue triangle points have been obtained from the spectrum of nanoparticles in mucus after subtraction of the mucus intensity contribution. (C) Intensity contribution of SVT-loaded PCL_P80 nanocapsules before (magenta dots) and after (blue triangles) interaction with mucus.

The SANS intensity profiles for blank and drug-loaded PCL_P80 nanocapsules (8 mg·mL^–1^) are reported in Figure 2A (colored symbols). In fact, the two spectra are superimposable and show a peculiar shape that has been reconstructed by a “raspberry” form factor (black lines), corresponding to particles made of globular units of markedly different sizes. The large oily core (120–160 nm) of PCL_P80 nanocapsules is stabilized by a shell of small globular aggregates, with size around 10 nm, resembling the surface of a raspberry.^59^ We observe that this formulation contains a high-volume fraction of the polysorbate 80 surfactant, bearing a highly hydrated hydrophilic headgroup, with three polyoxyethylene chains, which spontaneously form small micelles in an aqueous solution (size 9–10 nm). The SANS experiment suggests that a number of polysorbate 80 micelles might be adsorbed almost as such at the surface of the polymer shell surrounding the dispersed hydrophobic core. This is reasonable since the polysorbate 80 concentration in the preparation is well above the critical micellar concentration (13–15 mg·L^–1^ according to the product information of the supplier), and their overall effect is a stabilization of the structure of the nanoparticles (see the sketch in Figure 2A). These structural data are in agreement with results by Cé and co-workers, who observed such micelles in transmission electron microscope (TEM) images of dapsone-loaded PCL nanocapsules stabilized with polysorbate 80.^60^

The SANS exploration of the PCL_P80 nanocapsule interaction with the mucus model is reported in Figure 2B. The scattered intensity profile of drug-loaded PCL_P80 nanocapsules after 15 min interaction with the mucus model solution is shown as such (black diamonds) and after subtraction (blue triangles) of the intensity contribution of mucus (dark yellow). In Figure 2C, the subtracted experimental data are compared with the intensity profile of SVT-PCL_P80 before interaction with mucus. It can be appreciated that the two profiles are almost identical, revealing that no interaction occurred between the nanoparticles and mucus. This suggests that adhesion of mucin chains to the particles has been prevented by the external hydrophilic hairy shell of polyoxyethylene chains provided by the presence of polysorbate 80 micelles at the nanocapsule surface.

The steric stabilization and the resulting hampered glycoprotein adsorption at the nanoparticle surface induced by the hydrophilic coating (and poly(ethylene glycol) (PEG) chains in particular) are well known, and this is commonly exploited to produce “stealth” liposomes and nanoparticles with increased circulation lifetime after intravenous administration.^61,62^ Moreover, it has been proposed as a strategy to design mucus-penetrating nanoparticles, as suggested by investigating the nanoparticle mobility in mucus with the multiple particle tracking technique.^63,64^ Here, we report a structural confirmation that mucin chains do not interact or modify the structure of nanoparticles stabilized with pegylated surfactants, even if organized into a raspberry-like coating of micelles covering the polymeric nanoparticle core.

#### 3.2.3 Sodium Caproyl Hyaluronate-Coated PCL Nanoparticles

**Figure 3:**
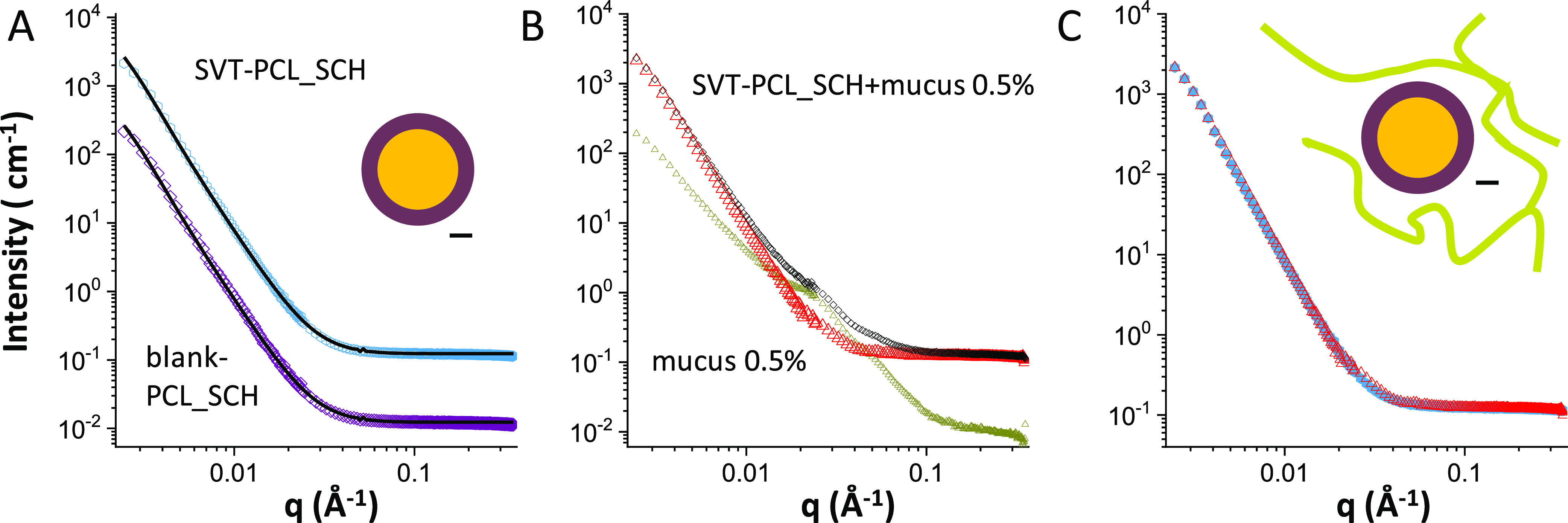
SANS intensity spectra of sodium caproyl hyaluronate-coated PCL nanocapsules (8 mg·mL^–1^). (A) Blank PCL_SCH (purple diamonds), shifted for better visibility, and SVT-PCL_SCH (cyan hexagon) nanocapsules together with the fit to a core–shell, as sketched. (B) Loaded SVT-PCL_SCH dispersed in simulated nasal mucus (porcine mucin 0.5% w/v in SNES) at 0.5% w/v (black diamonds) and simulated nasal mucus alonemucus at 0.5% w/v (dark-yellow circles). Red triangle points have been obtained from the spectrum of nanoparticles in mucus after subtraction of the mucus intensity contribution. (C) Intensity contribution of SVT-PCL_SCH nanocapsules before (cyan hexagon) and after (red triangles) interaction with mucus.

The intensity profiles, as measured by SANS, of SVT-PCL_SCH nanocapsules are reported in Figure 3A. The intensity decays have been modeled as a core–shell spherical form factor. Differently from LCN formulations, in the region ∼0.1 Å^–1^, spectra do not show any structure peak typical of multilamellar shells, indicating that the hydrophobic core of PCL nanocapsules (size larger than 200 nm in size) is stabilized by a single caproyl hyaluronate layer, which acts both as stabilizer and as a polymer for surface covering. Intensity spectra of SVT-loaded PCL_SCH nanocapsule interaction with the artificial mucus model are presented in Figure 3B.

After subtraction of the intensity contribution of mucus, the resulting profile is compared with the one of loaded nanoparticles before interaction with mucus (Figure 3C). The two profiles are superimposable, thus revealing no change in the nanoparticle structure. Results suggest that the presence of hyaluronate chains on the surface makes the particles stable also in the simulated nasal mucus model, despite the likely establishment of hydrogen and other noncovalent bonds with the mucus, which are attributed to hyaluronic acid and its derivatives.^65^ It is known that polymer-related factors, including molecular weight, chain flexibility, hydration, hydrogen-bonding capacity, and charge, can strongly modulate the mucin/polymer degree of interaction.^66^ We hypothesize that hampered adhesion between hyaluronic acid and mucin chains, both negatively charged, by electrostatic repulsion, is reinforced by hyaluronate functionalization with the fatty acid chains. The presence of hydrophobic lateral chains can affect the polymer chain flexibility, the swelling and organization in the aqueous layer surrounding the nanoparticles, and the polymeric binding capacity. Results correlating the chemical modification of hyaluronic acid (HA) to changes in mucoadhesion properties have been already reported.^67,68^ A reduced adhesion of HA to the ophthalmic mucosa after esterification of the polymer carboxylic groups was observed due to the reduced ability of the macromolecules to form hydrogen bonds.^68^ In addition, the anchoring of the hyaluronate derivative onto the polymeric core of the nanoparticle modifies the interfacial structure and the distribution of negative charges on the surface, key parameters in the bioadhesive properties of nanomaterials. Binding to the polymeric core, and reducing its chain mobility, may prevent the polysaccharide from entanglement and reduce the opportunity to form hydrogen bonds at the basis of hyaluronate mucoadhesion. The propensity of PCL_SCH nanocapsules to penetrate into the mucus matrix has also been corroborated by experiments on biological surfaces presented in the following sections.

### 3.3 *In Vitro* Cytotoxicity Assay of Nanoparticles

**Figure 4:**
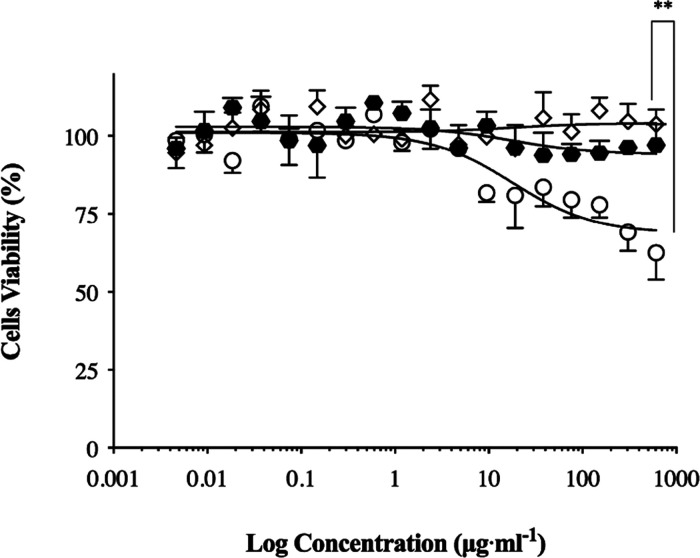
*In vitro* cytotoxicity studies on the human nasal cell line RPMI 2650 exposed to blank LCN (empty diamonds), blank-PCL_P80 (empty dots), and blank-PCL_SCH (black hexagons) nanoparticles. Cell viability after 72 h of incubation was calculated in comparison with untreated cells and is plotted against the logarithm of nanoparticle concentration, expressed as the total concentration of nanoparticle constituents in the medium (*n* = 3 independent experiments, **p < 0.01 compared to blank LCN).

The human RPMI 2650 epidermoid cell line derived from a nasal septum carcinoma is a suitable model of nasal mucosa to perform biological toxicity studies of formulations intended for nasal administration.^37^*In vitro* cytotoxicity assays were performed for all developed nanoparticulate carriers at several concentrations (from 0 to 610 μg·mL^–1^; concentration expressed as the total amount of nanoparticle constituent per unit volume of the medium). Viability of nanoparticle-treated cells at 72 h was recorded as percentage in comparison to untreated cells and is presented in Figure 4.

The cytotoxicity assay revealed that blank LCN and PCL_SCH nanoparticles were not toxic for all tested concentrations. On the other hand, 72 h treatment with blank PCL_P80 nanocapsules produced a decrease in cell viability at high concentrations, starting from 10 μg·mL^–1^. Extensive biocompatibility and toxicological investigations have shown that chitosan and hyaluronan derivatives are well tolerated when nasally administered,^69^ while nonionic surfactants, like polysorbate 80, have been related to some toxicity dependent on concentration and time of exposure.^70^ Still, the cytotoxicity assay (Figure 4) shows that PCL_P80 nanocapsule-treated cells maintained around 70% viability at 72 h even at the highest concentration tested. Since the average residence half-life in the nose is less than 45 min even for mucoadhesive formulations,^71^ we decided to move on in the investigation of the biopharmaceutical aspects for all three nanoparticle formulations here considered proposed.

### 3.4 Simvastatin Drug Release From Nanoparticles

To investigate another critical aspect of drug delivery systems designed for nasal delivery, drug release was evaluated *in vitro* through the dialysis bag diffusion method using a semipermeable cellulose membrane. SNES containing BSA at 0.5% w/v was used as the release medium to simulate the composition of the nasal secretions and to allow for sink conditions in a physiologically relevant medium, different from dissolution media containing surfactants and/or cosolvents, as previously reported.^22^ Although the SNES dissolution medium is simpler than the complex nasal secretions (containing mucus, proteins, enzymes, cellular and bacterial debris, etc.) and the time range of the experiment (24 h) exceeds the likely residence time of formulations in the nasal cavity, nevertheless the experimental setup allowed us to fully appreciate the different release kinetics of the three nanosystems and to compare them with the dissolution of the simvastatin raw material included as the control.

**Figure 5:**
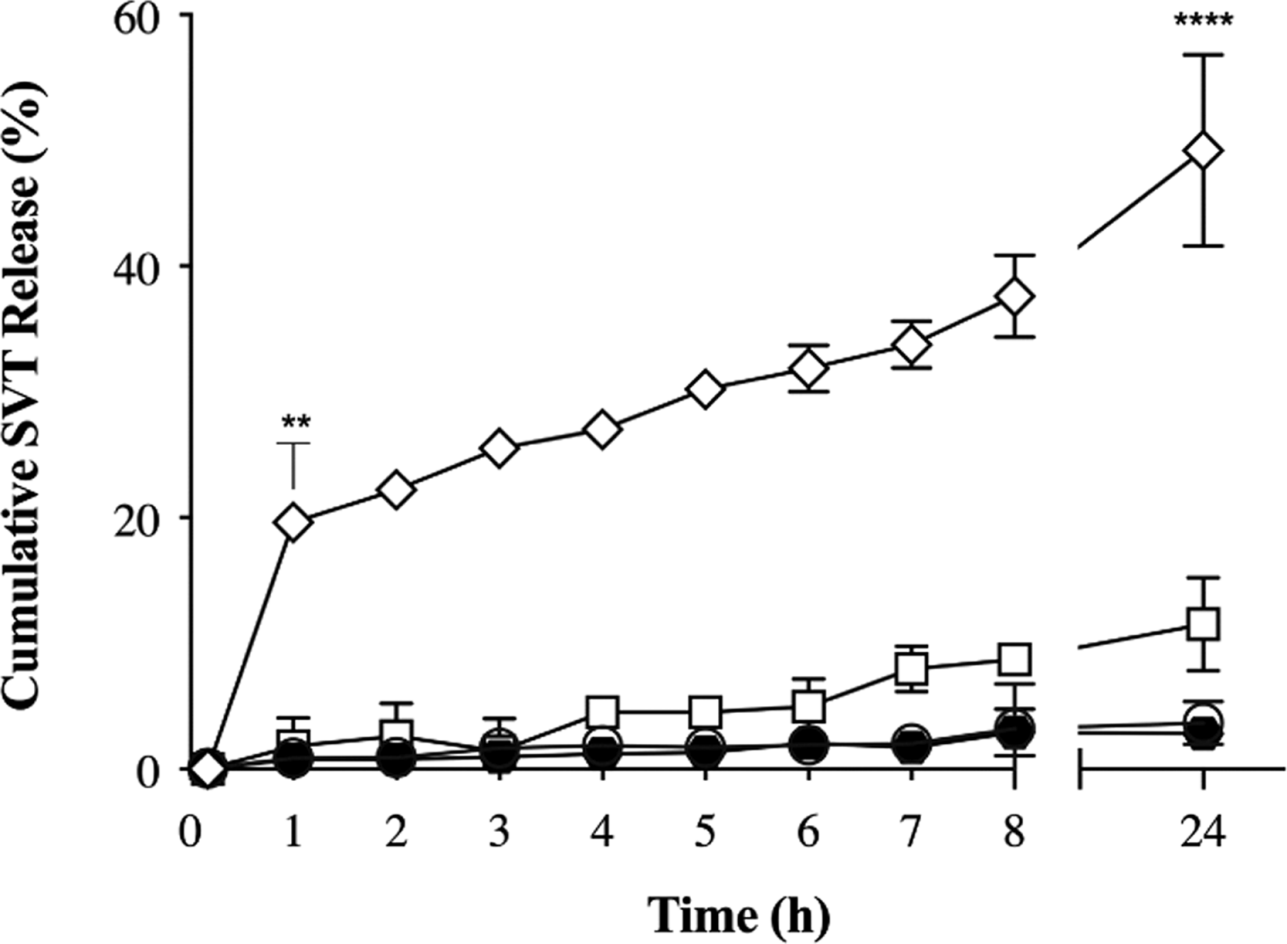
Cumulative *in vitro* drug release in SNES (pH 6.5 + 0.5% of BSA) from of the simvastatin suspension (empty square) and loaded SVT-LCN (empty diamonds), SVT-PCL_P80 (empty circles), and SVT-PCL_SCH (black hexagons) nanoparticles (*n* = 3, **p < 0.01 and ****p < 0.0001 compared to SVT-LCN).

In Figure 5, the cumulative drug-release profiles obtained from SVT-loaded nanoparticles and simvastatin suspension, as control, are presented. As compared to the raw material dissolution profile, SVT-loaded nanoparticles show two distinct and opposite behaviors. The release of simvastatin from SVT-PCL_P80 and SCH-coated SVT-PCL nanocapsules occurs at a constant but very slow rate, even slower than the dissolution of simvastatin from a simple suspension at the same concentration (1 mg·mL^–1^). In fact, both PCL-based nanocapsules strictly controlled the drug release to less than 5% within 24 h, as expected from their composition and nanostructure. In fact, it is well known that nanocapsules based on polyesters, like PCL, encapsulating the drug in their oily core and not adsorbed at least partially on the surface, ensure extremely slow release rates. Truly, the hydrophobic polymer shell acts as a physical barrier between the oily core and the release medium.^57,72^ On the other hand, SVT-LCN gave rise to a controlled but much faster drug diffusion process. Indeed, for SVT-LCN nanoparticles, an initial burst-release can be observed, with already 20% of the drug released in the first hour. In the following hours, the process slows down, reaching around 60% of the drug released in after 24 h. This behavior can be explained by the multilayered structure of the hydrophilic region of these nanoparticles, as assessed by SANS, with chitosan trapped within. Seemingly, the osmotic pressure generated in the hydrophilic domains of the nanoparticles upon contact with the release medium could favor the swelling of the polymer^73^ and the unwrapping of the outer layers, thus triggering drug release. Moreover, according to our previous work,^22^ in SVT-LCN nanoparticles, simvastatin appeared not only to be embedded in the oil core but also well dispersed in the shell structure. This structural feature can explain the observed initial burst in SVT release.

Generally, drug encapsulation in nanoparticles constitutes a strategy to either prolong the release at the site of absorption or at the target organ or provide permeation enhancement of the drug across biological barriers. We underline that for nasally administered formulations the mucociliary clearance is a major drawback, so that overall mucoadhesive but slow-releasing polymer nanoparticles are likely to be detrimental to the drug delivery process.^74^ However, an optimized performance can be attained by coupling strong mucoadhesiveness, retaining the drug formulation longer onto the nasal mucosa, to fast drug release, faster than the mucociliary clearance time scale. LCN nanoparticles present these features, so they are seemingly helpful to overcome nasal clearance and favor prompt drug absorption. Reversely, PCL_P80 and PCL_SCH, which display lower mucoadhesiveness and slow-release capacity, require to be taken up by cells to provide a significant improvement of intranasal drug delivery.

### 3.5 Nanoparticle Mucoadhesion on Excised Porcine Nasal Epithelium

The bioadhesion of nanoparticles with different surface features to a biological tissue was assessed by incubating the formulations onto the mucosal layer of porcine nasal tissue, followed by rinsing with SNES at a relatively high flow rate for 20 min (see also Figure S1). Differently from “static” permeation experiments, the experimental setup, including a continuous flow over the mucosal surface, aims at simulating, at least roughly, the nasal mucus turnover and removal of exogenous particles due to mucociliary clearance.^39^

**Figure 6:**
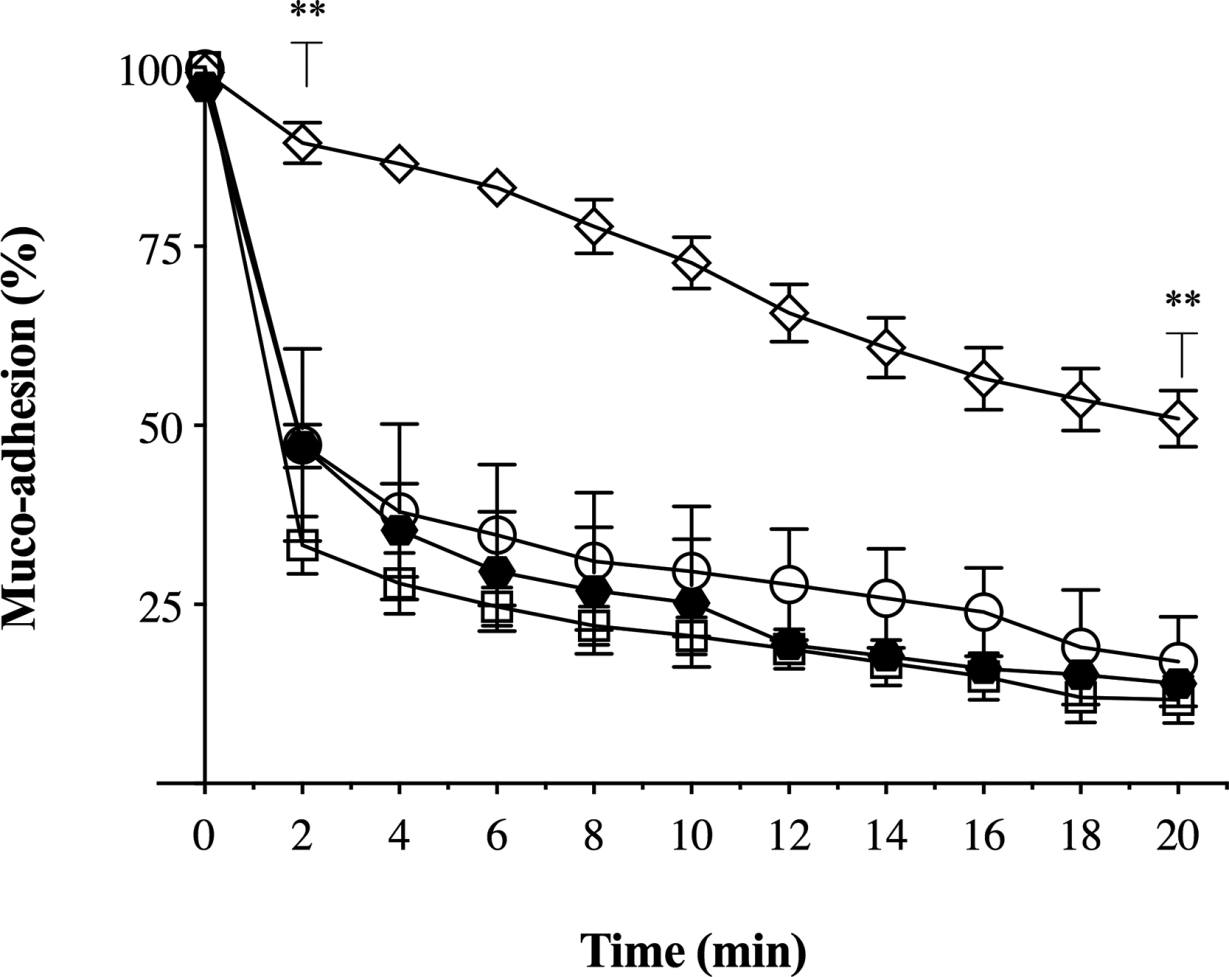
*In vitro* mucoadhesion of simvastatin suspension (empty square) and loaded SVT-LCN (empty diamonds), SVT-PCL_P80 (empty circles), and SVT-PCL_SCH (black hexagons) nanoparticles (*n* = 3, **p < 0.01 compared to SVT-LCN).

Figure 6 reports bioadhesion of the formulations tested expressed as a percentage of residual drug recovered from the epithelial nasal tissue over against time. Within 2 min of continuous washing, only half of the simvastatin deposited as SVT-PCL_P80 and SVT-PCL_SCH nanocapsules still adhered to the surface tissue layer (47.3 ± 18.9 and 47.1 ± 4.6%, respectively). Conversely, at the same short time delay, 90% of the SVT formulated in SVT-LCN was still found to adhere to the tissue. The chitosan-coated nanoparticles showed the strongest association with the nasal mucosa all along the experiment, with more than 50% (±2.30%) of SVT still stuck to the nasal mucosa after 20 min of continuous wash. In contrast, SVT-PCL_P80 and SCH-coated SVT-PCL allowed for only 15–20% residual simvastatin (17.1 ± 8.9 and 13.9 ± 2.5%, respectively), upon similar 20 min washing. Careful analysis inspection of data reveals that the removal rate was rather similar in the long period for the three formulations and that the major clearance for SVT-PCL_P80 and SCH-coated SVT-PCL occurred in the first 2 min. A similar profile was observed also for the simple drug suspension used as the control. In this case, the absence of suitable excipients promoting solubilization, mucoadhesion, or mucopenetration, the complete removal of SVT could be expected. Seemingly, simvastatin crystals (few micrometers in size) of the drug suspension were settled during the 5 min between the application and the start of the rinsing. The sedimentation into the tissue mucus layer then prevented the complete simvastatin removal even upon extensive rinsing. Considering all of these aspects, a mucosal mean residence time (mMRT) was calculated from continuous flow assay data, adapting the equations used for the well-known pharmacokinetic parameter MRT (see the Supporting Information). The mMRT of in SVT-LCN was found to be significantly higher than those of the other tested formulations (38.5 ± 1.9 min, *p* < 0.002). For the other formulations, the mMRT of SVT-PCL_SCH was found to be longer than the one calculated for the SVT-PCL_P80 nanoparticles and SVT suspension (19.4 ± 1.6 min vs 14.4 ± 3.5 and 14.7 ± 3.8 min, respectively) but not significantly different (*p* > 0.6).

Both bioadhesivity and SANS experiments evidenced that the chitosan-coated SVT-LCN nanoparticles undergo strong interaction with the mucous environment. Conversely, the hyaluronate-coated SVT-PCL_SCH nanocapsules displayed poor mucoadhesive properties. This is somehow surprising, given the mucoadhesivity currently attributed to hyaluronic acid and some derivatives, for several systems including ophthalmic,^75,76^ buccal,^66^ vaginal,^77,78^ gastrointestinal,^68^ and nasal mucosa.^51,65^ We concluded that in the mucoadhesion mechanism of polysaccharide-coated nanoparticles, a major role is played by electrostatic interactions. In fact, the sialic acid and ester sulfate residues from oligosaccharide chains of the mucin glycoproteins confer a high negative charge to the mucus,^79^ beyond viscosity and hydrophilicity. This disfavors negatively charged hyaluronate, while it promotes attractive interactions with chitosan, carrying positively charged amino groups, in agreement with what was observed in several works.^57,80^ Indeed, the variability of the physicochemical properties emerging from the complexity of nanoparticle composition, as well as the histological characteristic of tissues, makes it challenging to predict the performance of a drug delivery system in contact with the biological barrier, which is likely to depend as well on the mechanical evolution of the pair. Mucoadhesion is only a specific example of a more general phenomenon of adhesion, where the mucus covering the epithelial tissue concurs in the overall process of drug absorption and delivery. Moreover, we focused on the progressive washing interference due to continuous clearance, simulated as a constant force of shearing process, identified as a critical factor reducing the delivery performance for nasal application. We conclude that nasal delivery of the hydrophobic drug simvastatin could benefit from its formulation in chitosan-coated nanoparticles, allowing for an extended residence time at the site of application, e.g., the nasal cavity.

### 3.6 Simvastatin Transport across Excised Nasal Mucosa

The capability of nanoparticles to improve simvastatin transport across the nasal epithelium was assessed using mucosal tissues excised from the nasal septum of rabbits. Rabbit nasal mucosa was preferred over piglet nasal tissue because of the daily availability and due to the fact that a perfectly flat tissue, ideal for use with vertical diffusion cells, can be obtained from the septum.^41,81,82^ Indeed, a major concern for drug permeation and transport studies ex vivo is the nature and thickness of the tissue employed. The tissue of the nasal septum of rabbits is a preferred choice as a model for the investigation of biopharmaceutical aspects involving nasal drug delivery as it is relatively thin (from 50 μm to a maximum of 350 μm), covered by a pseudostratified columnar cell layer, has a ciliated respiratory epithelium, and allows for reproducible experiments.^83,84^

**Figure 7:**
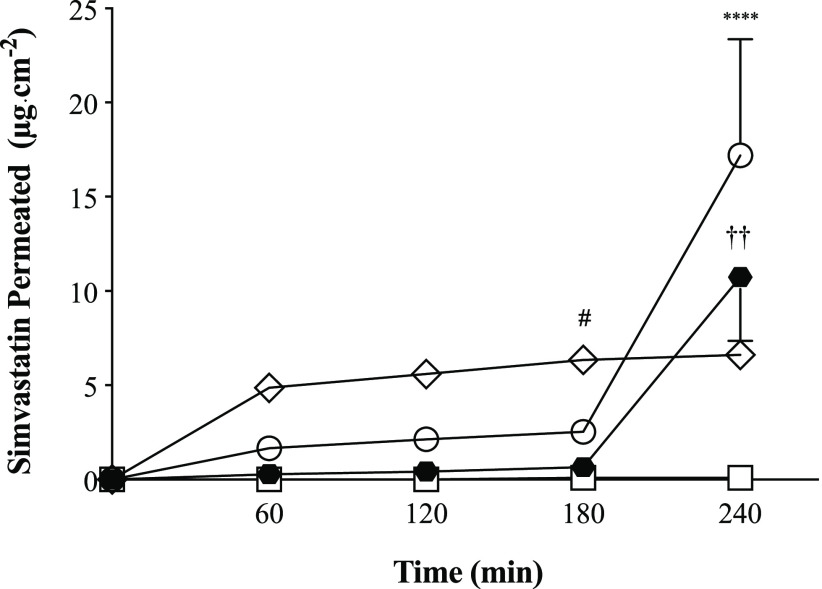
Profiles of simvastatin transport across the nasal epithelium of rabbit obtained for simvastatin suspension (empty square) and loaded SVT-LCN (empty diamonds), SVT-PCL_P80 (empty circles), and SVT-PCL_SCH (black hexagons) nanoparticles (*n* = 3, ^#^*p* < 0.05 for SVT-PCL_SCH compared to SVT-LCN; ^††^*p* < 0.01 for SVT-PCL_SCH compared to SVT-PCL_P80; ****p < 0.001 for SVT-PCL_P80 compared to SVT-LCN).

Transport profiles obtained for SVT-loaded LCN, PCL_P80 and PCL_SCH nanoparticles, compared to that obtained using a simple simvastatin suspension, reveal that the nanoparticle structure is critical in the nasal absorption of simvastatin (Figure 7). Indeed, encapsulation in nanoparticles enhanced the transport of simvastatin across the rabbit nasal epithelium when compared to the drug suspension. Actually, SVT permeation following application of the simple drug suspension was below the detection limit at all time points. The three nanoparticle formulations displayed two distinct permeation behaviors for simvastatin. Simvastatin permeation from chitosan-coated SVT-LCN nanoparticles was characterized by an initial strongly sustained simvastatin transport across the nasal mucosa, with ∼5 μg·cm^–2^ simvastatin transported in the first hour. In the same time interval, the permeation of simvastatin obtained with PCL_P80 and PCL_SCH nanocapsules was much lower (3-fold and 18-fold lower, respectively). After the first hour, the permeation profile obtained with SVT-LCN nanoparticles flattened considerably, nonetheless still keeping a positive slope, indicating a constant rate of drug diffusion, in accordance with the release kinetics observed in the *in vitro* experiments. After 4 h, the cumulative amount of simvastatin permeated by SVT-LCN nanoparticles per unit area of tissue was 6.63 ± 0.38 μg·cm^–2^. Conversely, SVT-loaded PCL_P80 and PCL_SCH nanocapsules presented a quite peculiar transport profile. In the case of SVT-PCL-SCH, simvastatin permeation was poor at the early time points and only allowed for only a negligible drug transport up to 3 h from the nanoparticles (0.66 μg·cm^–2^). In the case of SVT-PCL_P80 between 1 and 3 h, the transport profile showed a moderately positive slope, similar to the one provided by SVT-LCN in the same interval. Strikingly, after 4 h, a remarkable increase in transported simvastatin per unit area of tissue was observed for both PCL_P80 and PCL_SCH formulations, achieving 17.20 ± 6.15 and 10.74 ± 3.38 μg·cm^–2^, respectively. This indicates the onset of a different process driving drug permeation, beyond than drug release, triggered by the prolonged interaction between the nanoparticles and the nasal tissue.

To understand the nature of this interaction, at the end of the experiment, the nonpermeated formulation was removed, the external layer of the nasal tissue was thoroughly washed, and the amount of entrapped simvastatin was determined. The highest tissue accumulation of simvastatin (9.25 ± 5.79 μg) was provided by SVT-PCL_P80 nanocapsules, followed by SVT-PCL_SCH (7.27 ± 1.62 μg). Significantly lower amounts were found for SVT-LCN (2.89 ± 0.32 μg) and the control drug suspension (1.23 ± 0.29 μg). Taken together, these data suggest that along the whole process of transmucosal drug delivery, the nanoparticles under investigation take different complex pathways, each step being preferred, accelerated, or overridden according to the nanoparticle–substrate interaction and interference. It is possible that PCL-based nanocapsules are be actively taken up by cells via endocytosis because unrestrained by interaction with the mucus layer, they can slowly diffuse to the underlying cell surface.^85^

This could explain the values of simvastatin permeation observed for PCL_P80 and PCL_SCH only at longer incubation times. Intracellular nanoparticle accumulation may provide a steep gradient promoting delayed boosted transport resulting from transcellular transport of whole nanoparticles. On the other hand, since nanoparticles are entering the cells, the drug -release process may profit from the cell enzymes, which may trigger the nanoparticle structure degradation and modify the release kinetics. In fact, PCL nanocapsules do not release the drug *in vitro* but the hypothesis that intracellular biodegradation could provide the trigger for drug permeation has some experimental support in the literature. Namely, Barbieri and co-workers evidenced an increased transport of tamoxifen (another BCS class II molecule) across the intestinal epithelium of rats, about 6-fold and 24-fold upon addition of the enzymes pancreatin and lipase, respectively. The authors correlated the enzymatic biodegradation of the nanoparticles within the mucus layer, in the closest proximity of the epithelial cells, as the force driving the drug’s immediate availability and permeation.^46^ Using the same drug, Villemson and colleagues investigated the degradation of tamoxifen-loaded PCL nanoparticles in the presence of enzymes. While a very slow degradation is reported for PCL in the absence of enzymes (even years),^86,87^ these authors reported a rapid biodegradation kinetic of PCL nanoparticles in the presence of lipase, with the whole nanoparticle structure getting destroyed within 10 min.^88^ Interestingly, the SVT-LCN transport pathway seems to involve only marginally nanoparticle internalization and accumulation within the nasal epithelium, but rather proceed all along through gradient diffusion.

## 4 Discussion

The strong accumulation of simvastatin within the epithelial tissue promoted by its recruitment in PCL nanocapsules, as compared to the simple drug suspension and to chitosan-coated nanoparticles, suggests that these polymeric nanocapsules increased drug permeability through transcellular transport. Indeed, particles between 100 and 700 nm (and markedly around 100 nm)^89^ can be intracellularly transported through the nasal epithelium and, potentially, via the olfactory neural pathway to the brain.^90^ Only much smaller nanoparticles, less than 20 nm, appear to be able to exploit the extracellular transport, even when using absorption enhancers.^91^ Thus, size could not be considered the only factor affecting the different performance of the PCL-based nanocapsules with a similar size around 200 nm. Rather, the key factors determining their best performance are likely the composition and properties of their surface.

As for composition, PCL_P80 nanocapsules contain polysorbate 80, whose capability as an enhancer of nanoparticle permeability across nasal and respiratory biological barriers and of in vivo transport from the nose to the brain has already been demonstrated.^55^ The chemical structure and supramolecular organization of polysorbate 80 in micelles could play a key role in the behavior of PCL_P80 nanocapsules, increasing the thickness and hydration of the hydrophilic shell layer, promoting particle diffusion through the mucus, and uptake by cells.^92^ Indeed, polysorbate 80 was shown to be ineffective in promoting paracellular transport of hydrophilic drugs on human nasal epithelial cells (RPMI 2650 cell growth in air–liquid conditions), indicating that it does not induce the opening of nasal cells’ tight junctions.^93^ Similarly, the capability of SCH-coated PCL nanocapsules to foster SVT permeation, as compared to the simple drug suspension, is likely due to the presence of sodium caproyl hyaluronate as a surfactant. In fact, fatty acids themselves are described to increase the permeation of drugs across biological barriers, such as buccal and nasal^94^ mucosa, and their combination with a number of absorption enhancers, including hyaluronan derivatives, has been claimed to perform a synergistic action in improving nasal absorption of compounds, as compared to both promoters alone.^69^ Thus, the unique structure of the surfactant SCH, combining two synergistic permeation-enhancing excipients in a single ingredient, is likely to define the behavior of PCL_SCH nanocapsules in SVT delivery.

As for the surface charge of nanoparticles, it plays a crucial role in cellular adhesion and internalization. Indeed, although polysorbate 80 and sodium caproyl hyaluronate had proven to significantly enhance the absorption extent of simvastatin in the nasal epithelium, a lower extent of endocytosis was noticed for PCL_P80 and PCL_SCH, as often reported for negatively charged nanoparticles.^95^ Nevertheless, their effectiveness in crossing the nasal epithelium barrier, despite the repulsive force exerted by the negatively charged cell membrane, indicates that optimized nasal delivery by nanoparticles is played on their surface modulation well beyond mere electrostatic interactions.

Let us now turn to LCN nanoparticles, for which the role of chitosan as a permeation enhancer is well known.^96^ A key feature of this positively charged polysaccharide, evidenced also for chitosan-containing nanoparticles, is the combination of mucoadhesion and the capability to open tight junctions. Although fostered by this latter property, attributing the permeation enhancement observed for SVT-LCN to mere paracellular transport might be questionable, since one of the main limiting steps in the permeation of a BCS Class II molecule, like SVT, across the nasal mucosa, is the poor water solubility. Indeed, the drug accumulated within the tissue is similar for the control simvastatin suspension and SVT-LCN. However, the prompt and substantial drug release evidenced for SVT-LCN by *in vitro* experiments (see Figure 5) is likely to be the reason underlying the relatively rapid drug transport through the epithelial tissue, sustained effectively by a substantial drug concentration gradient across the mucosal barrier and enabled by a good permeability across biological membranes. In this process of SVT transport mediated by LCN nanoparticles, an important role is played by mucoadhesion, allowing for high tissue association as a consequence of the entrapment of drug nanocarriers within the overlaying mucus, as previously shown.^91^ Anchoring of chitosan may locally disturb the mucus network through the formation of bundles and the opening of pathways of faster diffusion, as already observed by our group in a mucus layer model.^33^ Moreover, within the mucus layer, the efficient release of the entrapped drug may further benefit from the presence of enzymes. In fact, our group has demonstrated in previous studies that also hybrid lecithin/chitosan nanoparticles can be biodegraded by nasal enzymes within the mucus barrier, providing a pivotal supplementary driving force in enhancing transcellular transport of lipophilic drugs.^21,46^ Those results can be attributed to the structure of hybrid lecithin/chitosan nanoparticles playing a decisive role as drug vehicles, enabling the transport of drugs across nasal barriers by exploiting nanoparticle interaction with the specific site of absorption to provide increased drug availability.

In conclusion, from a nasal drug delivery perspective, the produced nanoparticles can be classified according to their tactics in dealing with the mucosal barrier: mucoadhesion, pertinent to hybrid lecithin–chitosan nanoparticles, and mucopenetration, typical of PCL-based nanocapsules. In the polymeric PCL-based nanocapsules, mucopenetration is paralleled by high colloidal stability, in both SNES and mucus models, ensuring drug entrapment all along diffusion down to the underlying tissue. Here, the presence of synergistic permeation-enhancing components in the formulation sustains an efficient transport across the nasal epithelium. Thus, these features identify PCL-based nanocapsules as nanosized drug-carriers, probably promoting nasal absorption through the uptake of the whole nanocarrier encapsulating the drug. Reversely, hybrid lecithin/chitosan LCN nanoparticles behave as drug vehicles, strongly anchored within the nasal epithelial mucus layer and resisting/delaying mucociliary clearance. At the same time, the nanovehicle improves drug absorption by prompt drug release, triggered by a lower colloidal stability upon mucus contact and by the specific biodegradation carried out by enzymes present in the nasal secretions and tissues.^97^ Hence, the LCN nanoparticles do not carry the drug across the mucosal cell layer but enable an efficient absorption at the biological interface.

## 5 Conclusions

In this work, we explored three paradigmatic nanoparticles designed for nasal delivery of poorly water-soluble drugs, such as lipophilic statins, to efficiently increase their biological availability. Both putatively mucoadhesive (LCN chitosan-coated nanoparticles) and muco-penetrating (PCL hydrophilic polymer-coated nanocapsules) approaches were considered. Attention was focused on the impact of nanoparticle composition, structure, and surface properties on critical biopharmaceutical attributes, such as biocompatibility, drug release, mucoadhesion, and permeation across nasal tissues.

All three surface-modified nanoparticles showed excellent capacity to encapsulate simvastatin, without affecting their structural and surface properties, and displayed desirable features for nasal administration, such as biocompatibility, appropriate particle size, and high stability with elevated surface potential. In particular, in PCL-based nanocapsules, the stabilizing surfactant polysorbate 80 can be replaced with the novel polysaccharidic surfactant sodium caproyl hyaluronate, improving biocompatibility with human nasal cells, still preserving the main physicochemical properties of the nanocarrier.

The structural analysis highlighted however that the architecture of the three nanoparticles, dictated by their composition and manufacturing process, was definitely different, and it was determinant for their biopharmaceutical performance. In particular, drug release and mucoadhesion were substantially affected, ultimately determining alternative permeation pathways across nasal mucosa. Chitosan-coated LCN nanoparticles behave as mucoadhesive nanovehicles, prompting the absorption of the load at the interface through a rapid drug release, likely sustained by in situ biodegradation and enhanced permeation mediated by tight-junction opening promoted by chitosan. On the other hand, PCL-based nanocapsules, stabilized with polysorbate 80 or sodium caproyl hyaluronate, display very slow drug release and behave as mucus-penetrating nanocarriers. Hence, they need to be taken up by the epithelial cells to enable the crossing of the biological barrier.

In summary, by focusing on three paradigmatic nanoparticles, we highlighted that the modulation of the structure, surface, and physicochemical properties, via the careful selection of the components and the manufacturing methods, is a key strategy to optimize nasal and N2B delivery. In particular, the behavior at the biological interface of the nanomedicine can be blueprinted in terms of electrostatic interactions, mucoadhesion, permeation enhancement, and targeting of a specific absorption mechanism to direct the drug over the complex and limited transport pathways leading from the nose to the systemic circulation or even to the CNS, exploiting nose-to-brain transport.

In addition to the identification of the critical properties determining the biopharmaceutical fate of nanoparticles, the correlation between *in vitro* characteristics and *in vivo* bioavailability appears pivotal to select the most suitable nanostructure, if any, for the systemic nasal or nose-to-brain delivery of a given drug of interest. In fact, both types of delivery, the nanovehicle and the nanocarrier approach, present compelling features and potential drawbacks that have to be weighed up also in relation to the physicochemical properties, stability, mechanism of action, pharmacodynamics, and potency of the drug to be delivered.

## Supporting Information Available

Additional experimental details, materials, and methods, including schematic representation of the experimental setup (PDF)

## Abbreviations

BBB: blood–brain barrier
BCS: Biopharmaceutical Classification
CNS: central nervous system
DLS: dynamic light scattering
EE%: encapsulation efficiency
HPLC: high-performance liquid chromatography
LCN: lecithin/chitosan nanoparticles
MWCO: molecular weight cutoff
N2B: nose to brain
P80: polysorbate 80
PCL: poly-ε-caprolactone
PCL_P80: poly-ε-caprolactone nanocapsules stabilized with polysorbate 80
PCL_SCH: poly-ε-caprolactone nanocapsules stabilized with sodium caproyl hyaluronate
PI: polydispersity index
SANS: small-angle neutron scattering
SCH: sodium caproyl hyaluronate
SNES: simulated nasal electrolyte solution
SVT: simvastatin
ZP: ζ-potential
