# Supplementary material for: Structure and Fate of Nanoparticles Designed for the Nasal Delivery of Poorly Soluble Drugs
Source: Mol Pharm. 2021 Jul 14;18(8):3132–46. doi: 10.1021/acs.molpharmaceut.1c00366 (PMC8335725; doi:10.1021/acs.molpharmaceut.1c00366)
Supplement: Supplementary file 1 — mp1c00366_si_001.pdf [file mp1c00366_si_001.pdf]

Supporting Information

Methods

Nanoparticles preparation

Simvastatin-loaded lecithin/chitosan nanoparticles (SVT-LCN)

In detail, 50 mg of SVT were dissolved in 4 ml of 2.5% w/v lecithin alcoholic solution containing 100 mg each of Maisine™ and Labrafac™ oils. Following, the organic phase was injected using a glass pipette into 50 ml of 0.01% w/v chitosan aqueous solution, under constant magnetic stirring at 500 rpm and controlled temperature (40°C), for 15 min. Finally, ethanol was evaporated from the prepared colloidal suspension using a rotary evaporator (Heidolph WB/VV 2000, Schwabach, Germany) set at 40°C. Blank nanoparticles were produced as well, omitting simvastatin from the organic phase. All batches were prepared at least in triplicate and stocked for further physico-chemical and biopharmaceutical analysis.

Simvastatin-loaded PCL nanoparticles (SVT-PCL_P80 and SVT-PCL_SCH)

In detail, 20 mg of PCL, 10 mg of SVT, 24 mg of caprylic/capric triglyceride oil and 8 mg of sorbitan monostearate 60 were dissolved in 5 ml of acetone. Next, 1 ml of a previously prepared 0.06% w/v lecithin ethanol solution was added to the freshly prepared acetone solution to obtain nanoparticles organic phase. Formulation aqueous phase was obtained by dissolving 38 mg of polysorbate 80 into 10 ml of ultrapure water. SVT-PCL_P80 nanoparticles were then prepared following polymeric nanoprecipitation by injecting the organic phase, with the assistance of a glass pipette, into the aqueous solution, under moderate magnetic stirring (300 rpm) at 40°C. After 10 min stirring, the organic solvents were evaporated using a rotary evaporator (Heidolph WB/VV 2000, Schwabach, Germany) set at 40°C and formulation was further concentrated to a final volume of 10 ml.

Blank and SVT-loaded sodium caproyl hyaluronate (SVT-PCL_SCH) nanoparticles were obtained by same methodology. However, the aqueous phase contained replaced by 16 mg of the sodium caproyl hyaluronate acting as surfactant and stabilizer. All PCL-based nanoparticles were produced in triplicate, at least.

Nanoparticles Physicochemical Characterization

Nanoparticles Size and Zeta Potential Determination

Nanoparticle diameter, polydispersity index (PI) and zeta potential (ZP) were determined using a Malvern Zetasizer Nano ZSP (Malvern Instruments Ltd., Malvern, UK).

Nanoparticle diameter and PI were measured using dynamic light scattering (DLS) at 173° scattering angle. Prior to measurements, each formulation, *i.e.* blank and simvastatin-loaded nanoparticles, was diluted (1:100) with distilled water filtered with 0.22 µm filters (mixed cellulose esters membrane, Merck Millipore, Burlington, MA, USA), to avoid multiple scattering. For DLS measures, the instrument was set at 25 °C temperature and 173° scattering angle. Three measurements were recorded for all nanoparticles, in triplicate. ZP was determined through phase analysis light scattering (PALS) using the same diluted samples prepared for particle size analysis. ZP values are presented as mean and standard deviation of three separated runs for each sample prepared in triplicate.

Drug Encapsulation Efficiency of Nanoparticles

Nanoparticles SVT content and encapsulation efficiency (EE%) for SVT were evaluated by a direct and indirect method, respectively. EE% expressed as percentage of encapsulated drug with respect to the total amount present in the formulation. Briefly, the total amount of SVT (*Total SVT* in Eq.1) in each formulation batch was determined by dissolving 100 µl of SVT-loaded samples into 10 ml of standard diluent (ethanol: acetonitrile: water, 55:30:15, v/v/v, pH 4.5 adjusted with 1.0 M orthophosphoric acid), submitted to sonication (15 min, ultrasonic cleaner; VWR, Radnor, PA, USA), to favour the full release of the drug from nanoparticles. On the other hand, the free/non-encapsulated drug (Free SVT in Eq.1) present in the preparation was determined by ultrafiltration of 500 µl of each formulation in Vivaspin® centrifugal concentrator (PES membrane, MWCO 30 KDa, Sartorius, Gottingen, Germany; Medifuge, Heraeus Sepatech Gmbh, Hanau, Germany, 10 min, 4,000×g). The encapsulation efficiency of SVT in nanoparticles was then calculated using the following formula:

Eq. S1 $$EE\% = \frac{Total\ SVT - Free\ SVT}{Total\ SVT} \cdot 100$$

All quantification analyses were performed as three replicas for each formulation batch, prepared in triplicate, following the HPLC protocol illustrated in the following section.

High-Performance Liquid Chromatography Method for the Determination of SVT in Nanoparticles

A chromatographic system Agilent 1200 series (Agilent Technologies, Santa Clara, CA, USA), consisting in Agilent 1200 series UV-detector, auto-sampler, pump and a vacuum degasser unit was used. The chromatographic separation was carried out by gradient elution using a Symmetry Shield™ RP C8, 5 µm, 3.9 × 150 mm column (Waters Corp., Milford, MA, USA) as stationary phase, equilibrated at 45 °C. The mobile phase consisted of eluent A: ultra-filtered distilled water (cellulose acetate filter 0.22 µM, Sartorius, Gottingen, Germany), pH 5.5 adjusted with 1% of orthophosphoric acid and eluent B: methanol, applied at a constant flow rate of 1.5 ml/min. The eluting mobile phase composition started with 20 % of eluent B (0-2 min), increasing to 50% (2-4 min) and 80% (4-6 min) and held for 4 minutes at 100% (6-10 min), following the return to the initial conditions (20%) within 5 min for column equilibrium. UV-detector was set at 238 nm with sample injection of 20 µl. Detector signal processing was performed using Agilent ChemStation software (Rev B.03.02). The method was applied for the determination of both SVT isoforms, *i.e.* the lactone and hydroxyl acid form. Indeed, simvastatin hydroxyl acid isoform was obtained by the alkaline hydrolysis of the purchased lactone-form drug (for details see reference Clementino and Sonvico 2018) [17]. Linearity of calibration curves for both, SVT and its hydroxyl acid form, was verified in standard diluent (ethanol: acetonitrile: water adjusted at pH 4.5 with 1.0 M orthophosphoric acid, 55:30:15, v/v/v) in the range of 0.5–25 µg/ml (r^2= 0.9997 and r^2=_0.9996, respectively). Limit of detection and limit of quantification were 0.09 and 0.30 µg/ml for SVT and 0.10 and 0.28 µg/ml for the hydroxyl acid form.

Simvastatin Drug Release from Nanoparticles

In vitro release studies of SVT from drug-loaded LCN, PCL_P80 and PCL_SCH nanoparticles were carried out applying the dialysis bag diffusion method. SNES was used as dissolution medium since these nanoparticles are intended for nasal administration. Bovine serum albumin (BSA, 0.5% w/v) was used to increase SVT solubility in SNES (from 25 µg·ml^{-1} in to 52

$\mu g \cdot ml^{-1}$). For each formulation, 1 ml of the nanoparticle suspension (corresponding to approximately 1 mg of SVT) was dispersed into 1 ml of SNES + BSA 0.5% w/v, pH 6.5, to mimic nasal physiological conditions. Each 2 ml dispersion was separately placed in the dialysis tube membrane (MWCO 14 KDa, Sigma-Aldrich, St. Louis, MO, USA), in triplicate. The sealed bags were immersed into a graduated glass cylinder containing 100 ml of the dissolution medium (SNES 0.5% BSA), kept at 37°C and magnetically stirred at 100 rpm. At predetermined time points (1, 2, 3, 4, 5, 6, 7, 8 and 24 h), 500 µl aliquots of the dissolution medium were withdrawn from the cylinder. The sampled volume from each cylinder was replaced with an equal volume of fresh dissolution medium. Samples were then pre-treated with 25 µl of concentrated perchloric acid to precipitate and remove BSA by centrifugation (10 min at 21,380×g, D3024 Microcentrifuge, Scilogex, Rocky Hill, CN, USA). The obtained supernatants were four folds diluted in standard diluent (ethanol:acetonitrile:water adjusted to pH 4.5 with 1.0 M orthophosphoric acid, 55:30:15, v/v/v) and assayed by HPLC to quantify released SVT. Finally, to calculate SVT mass balance, the total content of each dialysis bag was quantitatively collected, dispersed into 50 ml of standard diluent and sonicated for 30 minutes to extract and quantify the residual drug from the nanoparticles formulations. All *in vitro* release studies were conducted in triplicate for each formulation and reported as percentage of drug released relative to from the total amount of simvastatin quantified in each experiment.

Nanoparticles Muco-adhesion on Excised Porcine Nasal Epithelium

The bio-adhesive strength of chitosan-coated LCN nanoparticles, PCL_P80 and SCH-coated-PCL nanoparticles were determined by Continuous Flow Assay, to evaluate the extent of adhesion/retention of a drug delivery systems on the surface of a mucosal tissue subjected to a controlled gravitational force and continuous wash.

First, porcine nasal tissues (discs, 6 mm in diameter) were excised from the nasal septum (obtained from spontaneously deceased piglets of local farms via the Department of Veterinary Medicine, University of Parma, Italy). The fresh piglet nasal mucosa discs were then put on a horizontal glass plate and kept in position by a small piece of double-sided adhesive. Then, 10 µl of either SVT-loaded LCN, or PCL_P80, or PCL_SCH nanoparticles or simvastatin aqueous suspension were applied, separately, on the nasal mucosa surface and left undisturbed for 5 minutes, allowing the samples to interact with the biological surface. The glass plate was then positioned upward on a polystyrene support oriented at a 45° angle

from the bench top and washing of the nasal mucosal surface with SNES was started at a constant flow rate (100 µl/min) for 20 min (see Fig. 1). The SNES flow was supplied by a syringe pump (Model 200, KD Scientific, Holliston, MA, USA). The nozzle tip was positioned perpendicularly to the surface of the glass plate, 3 mm distance from the tissue to ensure an even distribution of the liquid over the mucosal surface. Samples of the eluted washing SNES were collected every two minutes, diluted in standard diluent (1:1 v/v, ethanol: acetonitrile: water adjusted to pH 4.5 with 1.0 M orthophosphoric acid, 55:30:15, v/v/v) and assayed for SVT content by HPLC-UV, as reported in a previous section.

Nanoparticles Muco-adhesion on Excised Porcine Nasal Epithelium

Porcine nasal tissues (discs, 6 mm in diameter) were excised from the nasal septum (obtained from spontaneously deceased piglets of local farms via the Department of Veterinary Medicine, University of Parma, Italy). The fresh piglet nasal mucosa discs were then put on a horizontal glass plate and kept in position by a small piece of double-sided adhesive tape. Then, 10 µl of either SVT-loaded LCN, or PCL_P80, or PCL_SCH nanoparticles or simvastatin aqueous suspension were applied, separately, on the nasal mucosa surface and left undisturbed for 5 minutes, allowing the samples to interact with the biological surface. The glass plate was then positioned upward on a polystyrene support oriented at a 45° angle from the bench top and washing of the nasal mucosal surface with SNES was started at a constant flow rate (100 µl/min) for 20 min (syringe pump Model 200, KD Scientific, Holliston, MA, USA). The nozzle tip was positioned perpendicularly to the surface of the glass plate, 3 mm distance from the tissue to ensure an even distribution of the liquid over the mucosal surface. Samples of the eluted washing SNES were collected every two minutes, diluted in standard diluent (1:1 v/v, ethanol: acetonitrile: water adjusted to pH 4.5 with 1.0 M orthophosphoric acid, 55:30:15, v/v/v) and assayed for SVT content by HPLC-UV, as reported in a previous section.

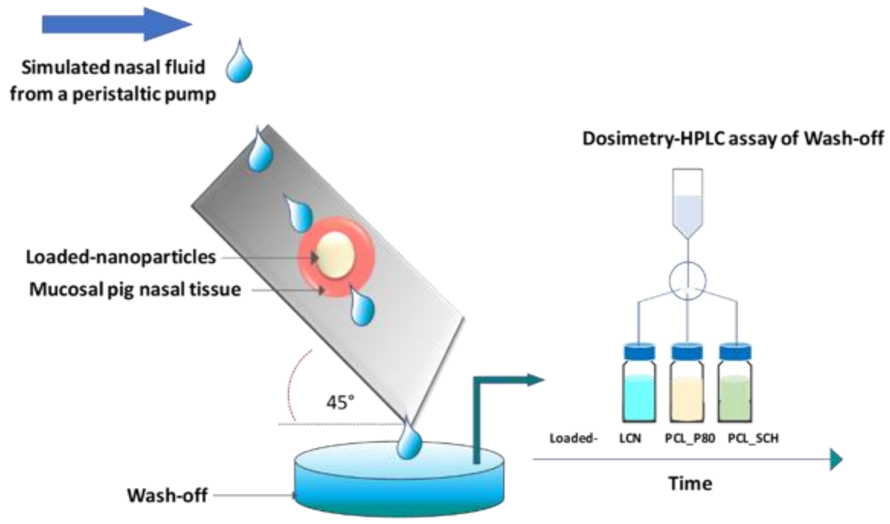

Figure S1. Schematic representation of the experimental settings for the in vitro studies of nanoparticles muco-adhesion strength.

At the end of the experiment, each nasal mucosa disc was collected and homogenized with 1 ml of standard diluent to extract and quantify the residual drug still present on mucosal surface. Results of the muco-adhesion analysis are reported as the amount of the residual simvastatin still adhering to the nasal mucosa at different washing times, expressed as percentage of the total amount recovered (cumulative amount of simvastatin collected in SNES washing samples plus the amount present on the mucosa at the end of the experiment). Mucosal mean residence time (mMRT) was calculated from the data collected, applying Equation S2, adapted from a classic method to calculate mean residence time in pharmacokinetics:[40]

$$\textbf{Eq. S2} \qquad mMRT = \frac{AUMC_{0-\infty}}{AUC_{0-\infty}},$$

where AUC is the area under the curve describing the percentage of residual SVT adhering to the tissue versus time and AUMC is the area under the first moment curve. The AUC and AUMC were calculated by the trapezoidal method and exponential extrapolation according to Equations S3 and S4:

$$\textbf{Eq. S3} \qquad AUC_{0-\infty} = AUC_{0-t_{last}} + \frac{M_{\%,last}}{k}$$

$$\textbf{Eq. S4} \qquad AUMC_{0-\infty} = AUMC_{0-t_{last}} + \frac{M_{\%,last} \cdot t_{last}}{k'} + \frac{M_{\%,last}}{k'^2},$$

where $AUC_{0\text{-}tlast}$ and $AUMC_{0\text{-}tlast}$ where calculated from data using the trapezoidal rule, $M_{\%,last}$ is the percentage of simvastatin adhering to the tissue at the last time point and k’ the

apparent elimination rate constant obtained as the terminal slope from the semilogarithmic graph of $M_{\%}$ vs. time.

Simvastatin Transport Across Excised Rabbit Nasal Mucosa

Rabbit heads were supplied by a local slaughterhouse (Pola S.r.l, Finale Emilia, Italy). Mucosal layers were excised from both sides of nasal septum within 2 hours from the animal's death. The specimens removed were rinsed with SNES (pH 6.5) and immediately mounted on vertical Franz-type diffusion cells (Vetrotecnica S.r.l., Padua, Italy; 0.58 cm^2 permeation area) with the mucosal side facing the donor compartment and serosal side facing the receptor.

For each cell, first, the donor compartment was filled with 500 µl of SNES solution and left for 5 minutes undisturbed, to check that no liquid was transferred to the receptor compartment due to inappropriate cell mounting or lack of tissue integrity. Then, the receptor chamber was filled with 5 ml of SNES (pH 6.5) and the assembled Franz cell was equilibrated at 37° C for half an hour in a thermostatic water bath prior to experiments. Thereafter, SNES solution was removed from the donor compartment and replaced with 1 ml of 1 mg/ml freshly prepared nanoparticles formulations, either SVT-loaded LCN, or -PCL_P80, or -PCL_SCH. The top of donor chamber was closed with Parafilm® (Sigma Aldrich Merck, Saint Louis, MO, USA) to prevent water evaporation. SVT suspension (1 mg/ml) in SNES was used as control. Experiments were carried out for 4 h, under constant magnetic stirring of the receptor compartment (800 rpm), to avoid boundary saturation on the mucosal membrane. At predetermined time points (0, 60, 120, 180 and 240 minutes), aliquots of 500 µl were sampled from the receptor compartment and replaced with the same volume of preheated SNES medium. Samples were kept at -20 °C until analysed.

At the end of the experiment, to calculate mass balance, donor samples were quantitatively collected, and the compartment rinsed thoroughly with SNES to recover any formulation residue adhered to glass walls or to the mucosal surface of the nasal tissue. Samples collected from donor were assayed for SVT content by dissolving 100 µl into 10 ml of acetonitrile: 25 mM PBS buffer (65:35 v/v, pH 4.5) and sonicating for 45 minutes (Ultrasonic cleaner; VWR, Radnor, PA, USA) to extract all the drug content from nanoparticles. Samples were filtered prior HPLC-UV analyses, to remove traces of precipitated proteins from the mucosal tissue. To extract and quantify the SVT content inside the mucosa, the same treatment described on

the previous section was applied. Data from SVT permeation were expressed as amount permeated per unit area ($\mu g \cdot cm^{-2}$) according to Equation S5,

$$\textbf{\textit{Eq. S5.}} \qquad Pe = \frac{Q}{A}$$

where *Pe* is the amount permeated per unit of area, *Q* is the total amount of drug transported (µg) and *A* is the diffusion area (cm^2).
